# Transcriptomic Profiling and Physiological Responses of Halophyte *Kochia sieversiana* Provide Insights into Salt Tolerance

**DOI:** 10.3389/fpls.2017.01985

**Published:** 2017-11-24

**Authors:** Long Zhao, Zongze Yang, Qiaobing Guo, Shun Mao, Shaoqiang Li, Fasheng Sun, Huan Wang, Chunwu Yang

**Affiliations:** ^1^Key Laboratory of Molecular Epigenetics of Ministry of Education, Northeast Normal University, Changchun, China; ^2^Department of Agronomy, Jilin Agricultural University, Changchun, China

**Keywords:** halophyte, RNAseq, Na^+^, water balance, root

## Abstract

Halophytes are remarkable plants that can tolerate extremely high-salinity conditions, and have different salinity tolerance mechanisms from those of glycophytic plants. In this work, we investigated the mechanisms of salinity tolerance of an extreme halophyte, *Kochia sieversiana* (Pall.) C. A. M, using RNA sequencing and physiological tests. The results showed that moderate salinity stimulated the growth and water uptake of *K. sieversiana* and, even under 480-mM salinity condition, *K. sieversiana* maintained an extremely high water content. This high water content may be a specific adaptive strategy of *K. sieversiana* to high salinity. The physiological analysis indicated that increasing succulence and great accumulations of sodium, alanine, sucrose, and maltose may be favorable to the water uptake and osmotic regulation of *K. sieversiana* under high-salinity stress. Transcriptome data indicated that some aquaporin genes and potassium (K^+^) transporter genes may be important for water uptake and ion balance, respectively, while different members of those gene families were employed under low- and high-salinity stresses. In addition, several aquaporin genes were up-regulated in low- but not high-salinity stressed roots. The highly expressed aquaporin genes may allow low-salinity stressed *K. sieversiana* plants to uptake more water than control plants. The leaf K^+^/root K^+^ ratio was enhanced under low- but not high-salinity stress, which suggested that low salinity might promote K^+^ transport from the roots to the shoots. Hence, we speculated that low salinity might allow *K. sieversiana* to uptake more water and transport more K^+^ from roots to shoots, increasing the growth rate of *K. sieversiana*.

## Introduction

Salinity has influenced agriculture in some areas for over 3,000 years (Jenks et al., 2007). In the last several decades, a great expansion of salinity-affected farmland and the rapid growth of the world's population have resulted in a serious threat to agriculture and food security worldwide (Jenks et al., 2007; Qadir et al., 2014). Although the breeding of salt-tolerant crops has been studied, no stronger salt-tolerant crops have been bred. The major reason is that plant salinity tolerance is a complicated phenomenon that involves morphological and developmental changes, as well as physiological and biochemical processes (Flowers and Colmer, 2008; Munns and Tester, 2008; Plett and Møller, 2010; Negrão et al., 2017). Salinity stress on plants can have multiple direct effects, including ion injury and osmotic imbalance, and indirect effects on soil structure and rhizosphere microbe populations (Munns and Tester, 2008). To survive in high-salinity environments, plants need not only to establish a complex regulatory network involved in most metabolic processes, but also to mediate the transport and partitioning of ions and organic solutes among organs or within cells (Flowers and Colmer, 2008; Munns and Tester, 2008; Plett and Møller, 2010). At the morphological level, some plants not only have special structural traits, such as, thick cuticles and salt glands, but also may need to change their biomass distribution (Läuchli and Lüttge, 2002; Tan et al., 2013). At the developmental level, plants may regulate their life cycles and even change their reproductive strategies (Maas and Poss, 1989; Khatun et al., 1995; Pushpavalli et al., 2016). Among the effects of salinity, osmotic stress produces more rapid damage than ionic stress, starting immediately after the salt concentration surrounding the roots increases to a threshold level (Munns, 2002; Munns and Tester, 2008); by contrast, ion injury exerts an more inhibitive effect on plant growth (Munns, 2002; Munns and Tester, 2008). To resist osmotic stress, plants usually synthesize organic solutes in the cytoplasm, such as, sugars, amino acids, and glycine betaine, and they accumulate inorganic ions in vacuoles (Rains, 1972; Hasegawa et al., 2000; Fricke, 2004; Kant et al., 2006; Flowers et al., 2010; Raven, 2010). Sodium (Na^+^)-stress tolerance in plants includes at least three processes: compartmentalization (at the cellular and/or tissue levels), exclusion (from shoots into roots or from roots into the rhizosphere), and long-distant transport (Munns and Tester, 2008; Plett and Møller, 2010; Suzuki et al., 2016a,b; Negrão et al., 2017). Several molecular mechanisms underlying salinity stress tolerance have been found in glycophytic plants, such as, rice, wheat, and Arabidopsis (Apse et al., 1999; Halfter et al., 2000; Shi et al., 2002; Sunarpi et al., 2005; James et al., 2006; Sahi et al., 2006; Brini et al., 2007; Horie et al., 2007; Garriga et al., 2016; Suzuki et al., 2016a,b; Zhang et al., 2016; Negrão et al., 2017). For example, *NHX* genes are responsible for Na^+^ compartmentalization in vacuoles; both the rice *HKT1;5* gene and wheat *HKT1;5* gene function in Na^+^ exclusion from shoots into roots; the Arabidopsis SOS system mediates Na^+^ exclusion into the rhizosphere (Munns and Tester, 2008; Plett and Møller, 2010). Several genes from some halophytes, such as, *Puccinellia tenuiflora, Salicornia brachiate*, and *Salicornia europaea*, have been reported to confer salinity tolerance of the transgenic glycophytes (Yadav et al., 2012; Liu et al., 2017). To date, however, the molecular mechanism of salinity tolerance of halophyte remains incompletely understood, because of the limited information of genomes and transcriptomes.

Most botanists believe that halophytes have different salinity tolerance mechanisms from those of glycophytic plants. Compared with glycophytic plants, halophytes prefer moderate-salt conditions rather than non-salt conditions (Khan et al., 2000; Flowers and Colmer, 2008; Flowers et al., 2010; Rozema and Schat, 2013). Indeed, some extreme halophytes show better growth under salinity conditions (even 400 mM NaCl) than without salt, while the same salinity would kill almost all glycophytic plants (Khan et al., 2000; Flowers and Colmer, 2008). Therefore, it is intriguing that why moderate salinity stimulates growth and that whether divergent mechanisms are employed for halophytes under moderate- and high- salinity stresses.

*Kochia sieversiana* (Pall.) C. A. M. is a special halophyte, and it is used as a forage grass, as well as a traditional Chinese medicinal material (Zhao et al., 2002; Yang et al., 2007). It can survive under extremely high-salinity stress and high-pH environments, even pH 11(Zhao et al., 2002; Yang et al., 2007). It often functions as a pioneer plant, invading heavily saline soils, and finally dominates the ecosystem. Here, we investigated its specific salinity tolerance mechanisms. For this purpose, the seedlings of *K. sieversiana* were treated with various concentrations of NaCl, and then physiological responses and transcriptome profiling of the stressed seedlings were surveyed. We measured contents of water and ions, biomass, 17 components of low molecular weight carbohydrates and 20 amino acids in the leaves of *K. sieversiana*. We particularly compared the expression responses of several significant salinity-tolerant gene families to moderate- and high-salinity stresses. We expected to make some connections between physiological responses and gene expression profiles, and then following questions would be addressed:

1. Does the adaptive mechanism of *K. sieversiana* differ under moderate- and high-salinity stresses?
2. Why does K. sieversiana show a better growth under moderate-salinity condition than under control condition?

## Materials and methods

### Plant materials and salt-stress treatments

Seeds of *K. sieversiana* (Pall.) C. A. M, which is not an endangered or protected species, were collected from the alkalized grasslands located in the west of Jilin Province, China. No specific permissions were required for seed collection. Seeds were planted in plastic pots containing thoroughly washed sand and placed in a greenhouse maintained at 25°C day and 18°C night under 16 h light. The plants were watered with half-strength Hoagland nutrient solution without NaCl daily for 30 d. To evaluate the salinity tolerance of *K. sieversiana*, the 30-d-old seedlings of *K. sieversiana* were treated with various concentrations of NaCl (0, 80, 160, 240, 320, 400, 480, 600, 800, and 1,000 mM) daily. The pots (15 plants per pot) were watered thoroughly once every day between 17:00 and 18:00 h with the application of nutrient solutions containing the appropriate concentration of NaCl. Control plants were maintained by watering with nutrient solution. When the seedlings were stressed for 48 h, RNA samples were collected, and at 30 d physiological measurements were taken.

### Biochemical measurements

After the seedlings were stressed with 0, 80, and 480 mM of NaCl for 30 d, samples were harvested and freeze-dried for biomass determination and biochemical analyses. Five seedlings were pooled as a biological replicate. For each treatment, four replicates were used for the biochemical measurements. Tissue powders (0.1 g) were digested with HNO_3_ at 120°C. The content of mineral elements, including Ca^2+^, K^+^, Fe^3+^, Mg^2+^, Na^+^, and P^5+^, were measured using an inductively coupled plasma emission spectrometer. Selective transport capacity of ion (ST) was calculated using following formula: (root Na^+^/K^+^)/(leaf Na^+^/K^+^). The greater ST value shows stronger capacity that root promotes K^+^ to transport to leaf (Wang et al., 2002, 2009).

Amino acids and carbohydrates of freeze-dried samples were measured with a high-performance liquid chromatograph (HPLC) linked to a triple quadrupole/linear ion trap mass spectrometer (MS; QTRAP 3200, Thermo Scientific, Germany). Amino acids of samples were extracted using distilled water at 50°C. Amino acids were treated with a derivatization kit (Puruihuasheng Biological Science And Technology Limited Company Beijing, China) and were then analyzed using an HPLC equipped with a MSLab 45+AA-C18 (150 × 4.6 mm, 5 μm) column, linked to a triple quadrupole/linear ion trap mass spectrometer system with an electrospray ionization source (ESI;QTRAP 3200, Thermo Scientific). Formic acid (0.1%, v/v) and acetonitrile with 0.1% (v/v) of formic acid were employed as mobile phases A and B, respectively. The column temperature was set at 50°C, and the flow rate was 1 mL/min. The MS conditions were as follows: source: ESI (positive ion mode); needle voltage: 5,500 V; source temperature: 500°C; gas: nitrogen; nebulizing gas: 55 psi; focusing gas: 60 psi; scan type: MRM.

Carbohydrates of freeze-dried samples were extracted using distilled water at 50°C and were then analyzed using an HPLC equipped with an innoval-NH2 (250 × 4.6 mm, 5 μm) column linked to a triple quadrupole/linear ion trap MS system with anESI (QTRAP 3200, Thermo Scientific). Water (0.2 mL/min) and acetonitrile (0.8 mL/min) were employed as mobile phases A and B, respectively. The column temperature was set at 30°C. The MS conditions were as follows: source: ESI (positive ion modes); needle voltage: −4,500 V; source temperature: 500°C; gas: nitrogen; nebulizing gas: 50 psi; focusing gas: 60 psi; scan type: MRM.

### RNA isolation, DNA library preparation, and sequencing

After the seedlings were stressed with 0, 80, and 480 mM NaCl for 48 h, samples of leaves and roots were collected and immediately frozen in liquid nitrogen. There were three biological replicates per tissue for one treatment, and each biological replicate involved five plants for pooling. Total RNA was extracted from these frozen tissues using TRIzol reagent (Invitrogen). A NanoDrop ND2000 spectrophotometer and an Agilent 2100 Bioanalyzer were used to examine the quantity and quality of the total RNA. Library construction, cluster generation, and HiSeq 2000 sequencing were performed according to standard protocols. For samples to be treated with experimental parallelism and examined by the same HiSeq 2000 run, the raw data were filtered with an NGS toolkit(http://www.nipgr.res.in/ngsqctoolkit.html) with 85% above Q30 (Table 1) and stated by FastaQC (http://www.bioinformatics.babraham.ac.uk/projects/fastqc/).

**Table 1:** Overview of the differentially expressed unigenes of *K. sieversiana*.

	**Total trinity “genes”**	**Total trinity transcripts**	**Percent GC (%)**	**Contigs N50 (bp)**	**Median contigs length (bp)**	**Average contigs length (bp)**	**Total assembled bases**
Original sequence	469,047	597,278	42.2	1,057	382	684.45	408,807,562
Final sequence	47,965	47,965	41.48	1,935	1,174	1513.32	72,586,503

### *De novo* assembly and identification of differentially expressed genes (DEGs)

We used conventional methods and software for the *de novo* assembly and identification of DEGs. Trinity (https://github.com/trinityrnaseq/trinityrnaseq/wiki; Grabherr et al., 2011) was used to obtain the *de novo* assembly of the clean reads, and then the fragments per kilobase of transcript per million mapped reads (FPKM) for each unigene were computed to determine the unigene expression profiles using the RSEM (https://github.com/deweylab/RSEM) with a reference that was the aforementioned result of Trinity (Li and Dewey, 2011). Next, we identified unigenes that were diversely expressed among the three treatments. Unigenes with numbers of counts more than 50 and with no blanks, were selected for the following analyses. A DEGs analysis was performed using a perl script provided by Trinity to call edgeR, and the data matrix of log_2_ Fold Change, log_2_-counts-per-million, *P*-values, as well as false discovery rates (FDRs) were produced. DEGs were calculated between salinity levels of 0 and 80 mM, 0 and 480 mM, as well as 80 and 480 mM in leaf and root samples. Genes with an FDR threshold ≤ 0.05 and fold change ≥ 2 (absolute value of log_2_ Fold Change ≥ 1) were removed from the data matrix files generated by the aforementioned step by another perl script provided by Trinity. Open reading frames (ORFs) were predicted by TransDecoder (http://transdecoder.github.io/) using the Pfam domain search method.

### DEGs were validated using qRT-PCR

*UBQ10* was used as the normalization control gene in the assay (Jian et al., 2008). The primer sequences are listed in Table S1, and the expression levels were calculated using the ΔCt method (Livak and Schmittgen, 2001).

### Functional annotation

The protein sequences generated by TransDecoder were queried using a BLASTP algorithm-based search (*E*-value < 1e-5) of the public proteins databases, including the National Center for Biotechnology Information (NCBI)'s non-redundant protein database (Nr), Swiss-Prot, Cluster of Orthologous Groups of proteins (COG), Conserved Domain Database (CDD), Gene Ontology (GO), as well as Kyoto Encyclopedia of Genes and Genomes Database (KEGG). The Nr database was downloaded from NCBI (ftp://ftp.ncbi.nih.gov/blast/db/), and the Swiss-Prot database was downloaded from uniprot (ftp://ftp.uniprot.org/pub/databases/uniprot/current_release/knowledgebase/complete/uniprot_sprot.fasta.gz) for more accurate protein annotations. The COG database (ftp://ftp.ncbi.nih.gov/pub/COG/KOG/kyva) was downloaded for COG annotation and classing. CDD results were produced through NCBI (http://www.ncbi.nlm.nih.gov/Structure/bwrpsb/bwrpsb.cgi). The GO annotation and enrichment analysis of DEGs within the aforementioned DEG matrix data were performed using the Blast2GO program (https://www.blast2go.com/blast2go-pro). We submitted protein sequences to KEGG (http://www.genome.jp/kaas-bin/kaas_main) for annotation and then enrich them using R (https://www.r-project.org/) using the hypergeometric distribution model.

### Statistical analysis

Statistical analysis was performed using the R and SPSS software. For physiological data, mean of different treatments were compared by least significant difference test (*P* ≤ 0.05) by SPSS. For bioinformatic data, edgeR software was employed to identify DEGs. Genes with an FDR threshold ≤ 0.05 and fold change ≥ 2 (absolute value of log_2_ Fold Change ≥ 1) were considered as DEGs.

## Results

### *De novo* assembly and functional annotation of the *K. Sieversiana* sequences

We measured three biological replicates per treatment (salinity levels of 0, 80, and 480 mM), and each biological replicate was a pool of five plants. We collected 618,744,848 raw reads using Illumina HiSeq2000. Furthermore, we filtered raw data and obtained 502,491,351 clean reads for *de novo* assembly. We conducted a *de novo* assembly using Trinity software, and produced 469,047 unigenes. The contigs' N50 was 1,057 bp, and the GC content was 42.20%. We further selected the high quality unigenes with number of counts >50 and no blanks, and the resulting 47,965 unigenes had an N50 of 1,935 bp and a 41.48% GC content (Table 1). We also performed an ORF analysis of the 47,965 unigenes, and 46,824 unigenes (97.6%) had ORFs containing start codons.

The 46,824 high-quality unigenes were used as query in BLAST-algorithm-based searches against Nr, Swiss-Prot, COG, CDD, GO, and KEGG databases. In total, 38,686 (~83%) and 29,309 (~63%) of unigenes were matched to Nr and Swiss-Prot databases, respectively, indicating that the quality of the sequences was high (Figure S1, Table S2). Additionally, 3621 DEGs of leaf samples were enriched on 142 KEGG pathways and 55 GO terms, and 3012 DEGs of root samples were enriched on 161 KEGG pathways and 50 GO terms (Table S3).

### Calculation of DEGs and genes regulated by salt stress

**Figure 1:**
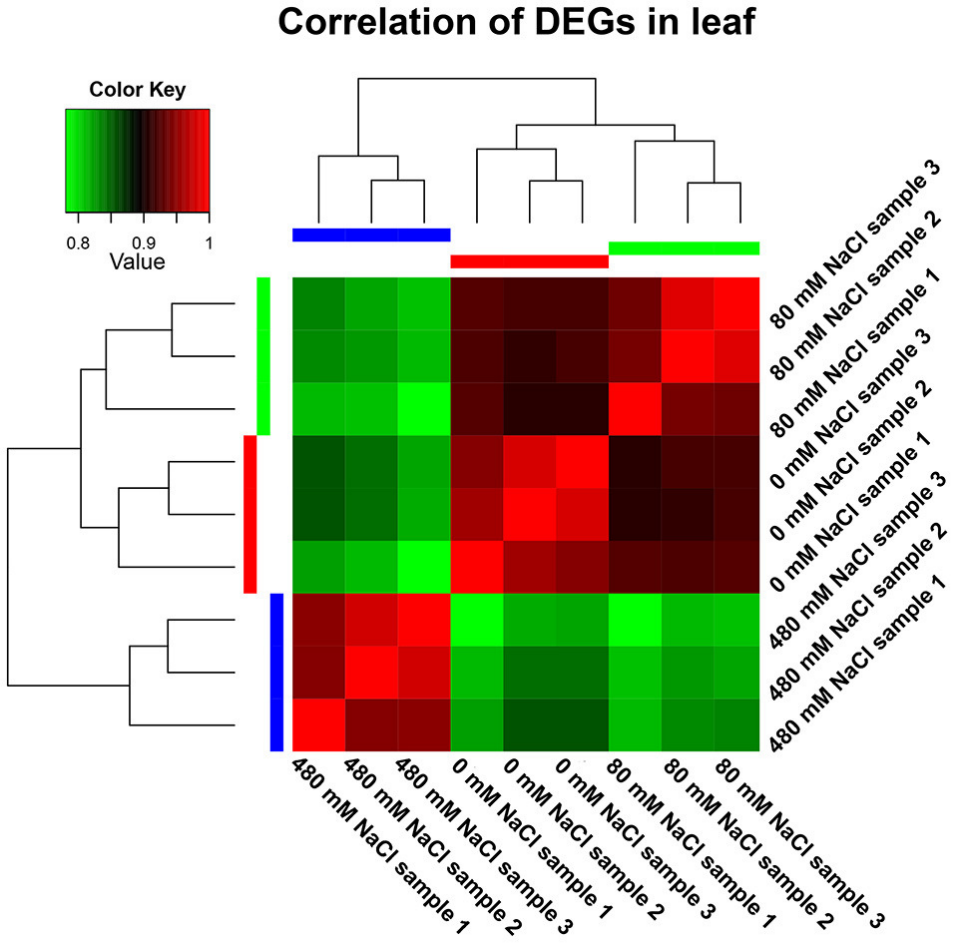
Correlation analyses of gene expression from nine leaf samples. The 30-d-old seedlings were subjected to 0, 80, and 480 mM NaCl for 2 d.

We analyzed the expressional changes of all *K. sieversiana* genes in response to salinity stresses, and found 3012 DEGs in the leaves and 3621 DEGs in the roots (FDR threshold ≤ 0.01 and fold change ≥ 2; Figure S2). Additionally, in the leaves, the number of DEGs between control and high-salinity stress was 8.3-fold that between control and low-salinity stress and in the roots it was 1.7-fold (Table 2). We also surveyed DEGs between low- and high-salinity treatments, and found many DEGs. Moreover, the correlation between the low-salinity treatment and control is greater than that between the high-salinity treatment and control (Figure 1, Figure S3).

**Table 2:** Overview of differentially expressed unigenes in leaves and roots of *K. sieversiana*.

	0 > 80^*^	**0 < 80**	**0 > 480**	**0 < 480**	**80 > 480**	**80 < 480**	**Sum**
Leaf	155	100	1,078	1,032	1,298	659	3,012
Root	705	474	886	1,102	1,082	1,171	3,621

* *“0 > 80” stands for number of up-regulated genes under 0 mM NaCl compared with 80 mM NaCl, and so on*.

**Figure 2:**
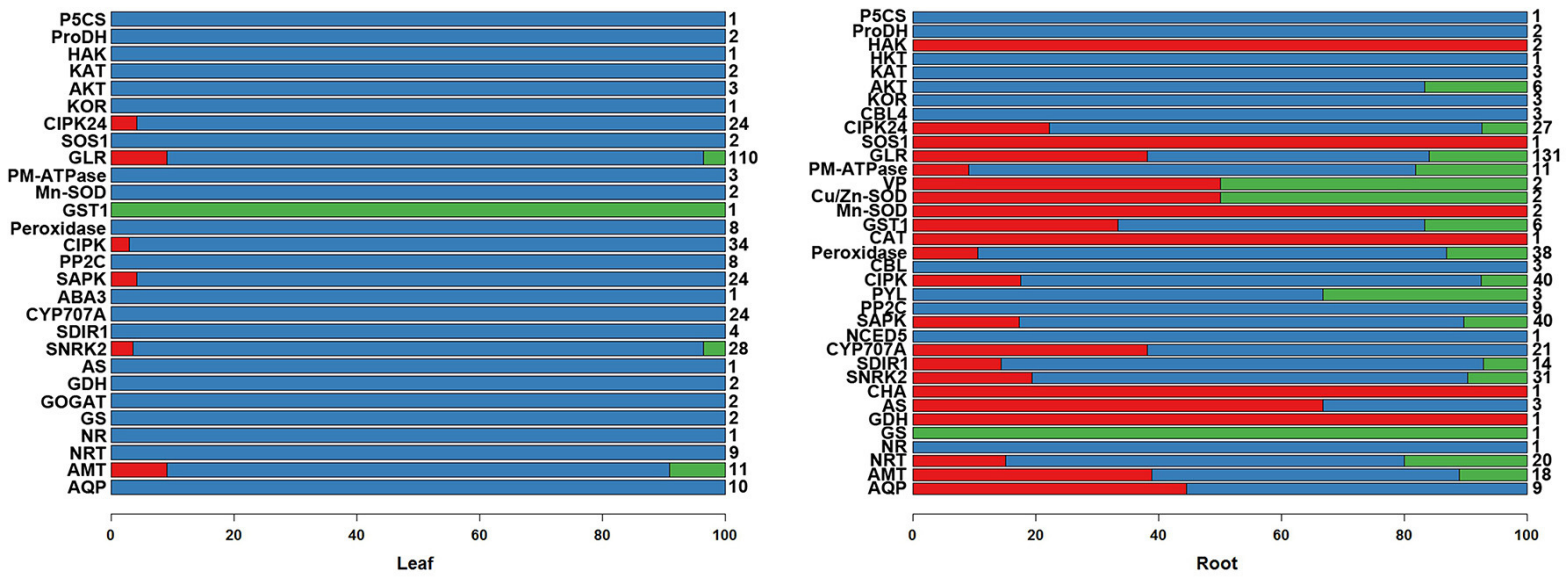
The percentages of various response patterns of salinity-tolerant gene families to salinity stresses in the leaves and roots of *K. sieversiana*. Only differentially expressed genes were considered in the column diagram. The different members of each salinity-tolerant gene family were grouped into three response patterns: the expression was changed by only 80 mM NaCl (red); only by 480 mM NaCl (blue); by both 80 and 480 mM NaCl (green). Total member numbers of the gene families are listed on the right of the column diagram. The 30-d-old seedlings were subjected to 0, 80, and 480 mM NaCl for 2 d.

We further analyzed the DEGs of 36 gene families involved in the salinity tolerance mechanism (Figure 2, Figures S4–S6). We were particularly interested in the differential expression response patterns (The red: gene expression was only changed by 80 mM NaCl; blue: only by 480 mM NaCl; green: by both 80 and 480 mM NaCl; Figure 2). The expressional change trends of these salinity tolerance gene families were similar to the above results of the genome-wide DEGs. In the leaves, rare DEGs were observed under 80-mM NaCl stress (red), while abundant DEGs were found under 480-mM NaCl stress. Similarly, in the roots, DEGs relatively more frequently existed under 480-mM NaCl stress. Based on our DEG and correlation analyses, we propose that the cellular environments of *K. sieversiana* were mildly disrupted by low salinity but greatly by high salinity, which may result in the activation of different genes in the resistance to low-and high-salinity stresses. In addition, we only found one differentially expressed *HKT* gene and *SOS1* gene, and zero *NHX* gene that were principal for glycophytic plants to resist salt-stress. Thus, we propose that some classically important salt tolerance genes in glycophytic plants may play novel roles or no roles in *K. sieversiana*. We validated the results of 10 genes using qRT-PCR. We obtained the similar results to RNAseq (Table S4).

### Growth and oxidative stress

**Figure 3:**
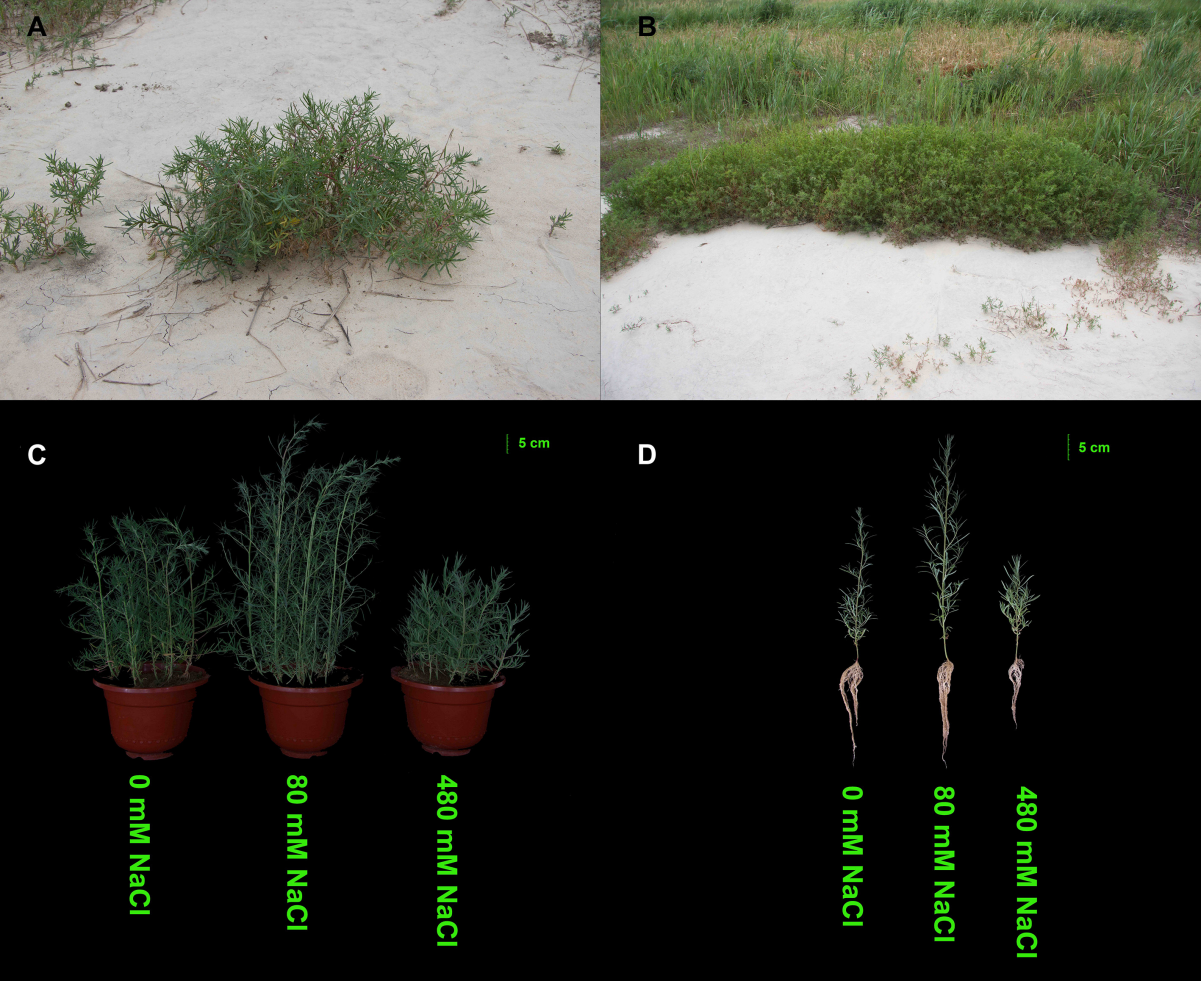
Effects of salinity stress on the growth of *K. sieversiana*. **(A,B)** Growth status of *K. sieversiana* in its natural saline–alkaline habit. **(C,D)** Effects of various concentrations of NaCl on the growth of *K. sieversiana*. The 30-d-old *K. sieversiana* seedlings were treated with various concentrations (0, 80, and 480 mM) of NaCl for 30 d. The growth status of *K. sieversiana* under NaCl stresses.

**Figure 4:**
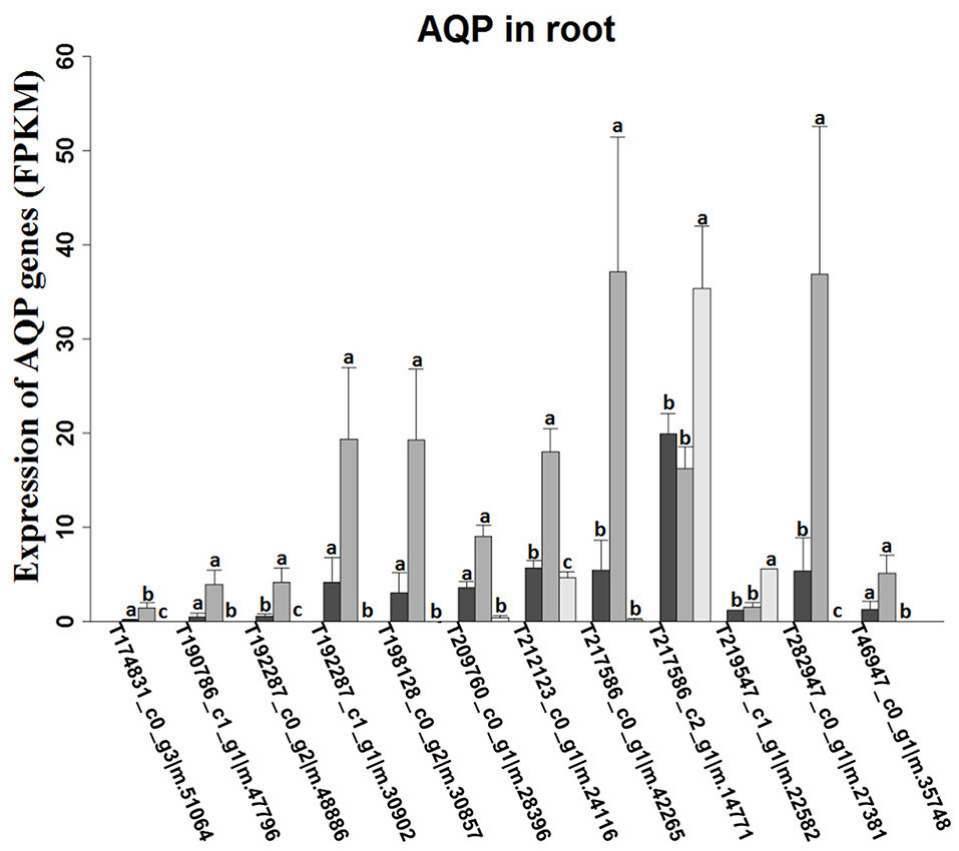
Expression levels of aquaporin-like unigenes in roots of *K. sieversiana* under different salinity treatments. The values are means of three replicates (±SE). The 30-d-old seedlings were subjected to 0, 80, and 480 mM NaCl for 2 d. Different letters above the bars indicated significant differences among treatments, according to a least significant difference test (*P* ≦ 0.05).

*K. sieversiana* has a competitive advantage over other plants in extremely saline grasslands and maintains noticeable biomass and remarkable growth (Figure 3). In this study, to investigate its specific salinity tolerance mechanisms, we treated plants with 0–1,000 mM NaCl. *K. sieversiana* can grow well under 800 mM NaCl. We further measured five growth indices and 44 biochemical indices of *K. sieversiana* only under 0, 80, and 480 mM NaCl. Under low-salinity stress (80 mM NaCl) *K. sieversiana* showed better growth than under control conditions, and 80-mM NaCl-stressed plants had greater values for biomass, fresh weight, water content (WC), and stem diameter (Table 3, Figure S7). The WC increased with increasing salinity. To find the possible molecular mechanisms behind the high WC of this halophyte, we analyzed the aquaporin (*AQP*) genes' expression data from the RNAseq data. In the leaves, the expression levels of most *APQ* genes under the 80-mM NaCl treatment remained unchanged compared with control treatment, but all those under the 480-mM NaCl treatment were declined (Figure S8). In roots, 10 up-regulated *AQP* genes were found under low-salinity stress, while only two up-regulated *AQP* genes were observed under high-salinity stress (Figure 4). We also analyzed the data of the genes involved in oxidative stress tolerance (Figures S5, S6, S9). A number of DEGs were observed in *peroxidaxe* gene family, but few DEGs were found in *Cu/Zn-SOD, CAT, Mn-SOD*, and *GST1* families and no DEG in *GPX* (Figure S9). The result showed that *Mn-SOD* gene was up-regulated in high-salinity stressed leaves, that a *Cu/Zn SOD* gene was up-regulated in low-salinity stressed roots but not in high-salinity, that the expression of a *CAT* gene was enhanced in low-salinity stressed roots, and that several *GST1* and *peroxidaxe* genes were up-regulated either under high- or low-salinity stress (Figures S5, S6, S9).

**Table 3:** Effects of salinity stress on the growth of *K. sieversiana*.

**Salinity (mM)**	**Fresh weight (g plant^−1^)**	**Dry weight (g plant^−1^)**	**Water content (g g^−1^ DW)**
0	3.97 ± 0.30^b^	0.83 ± 0.07^b^	3.78 ± 0.07^c^
80	5.70 ± 0.44^a^	1.02 ± 0.06^a^	4.56 ± 0.15^b^
480	3.28 ± 0.15^b^	0.46 ± 0.03^c^	6.22 ± 0.24^a^

*The values are means ± SEs of four replicates. Different letters above the bars indicate significant differences among treatments according to a least significant difference test (P ≦ 0.05).The 30-d-old seedlings were subjected to 0, 80, and 480mM NaCl for 30 d*.

### Ion balance

**Figure 5:**
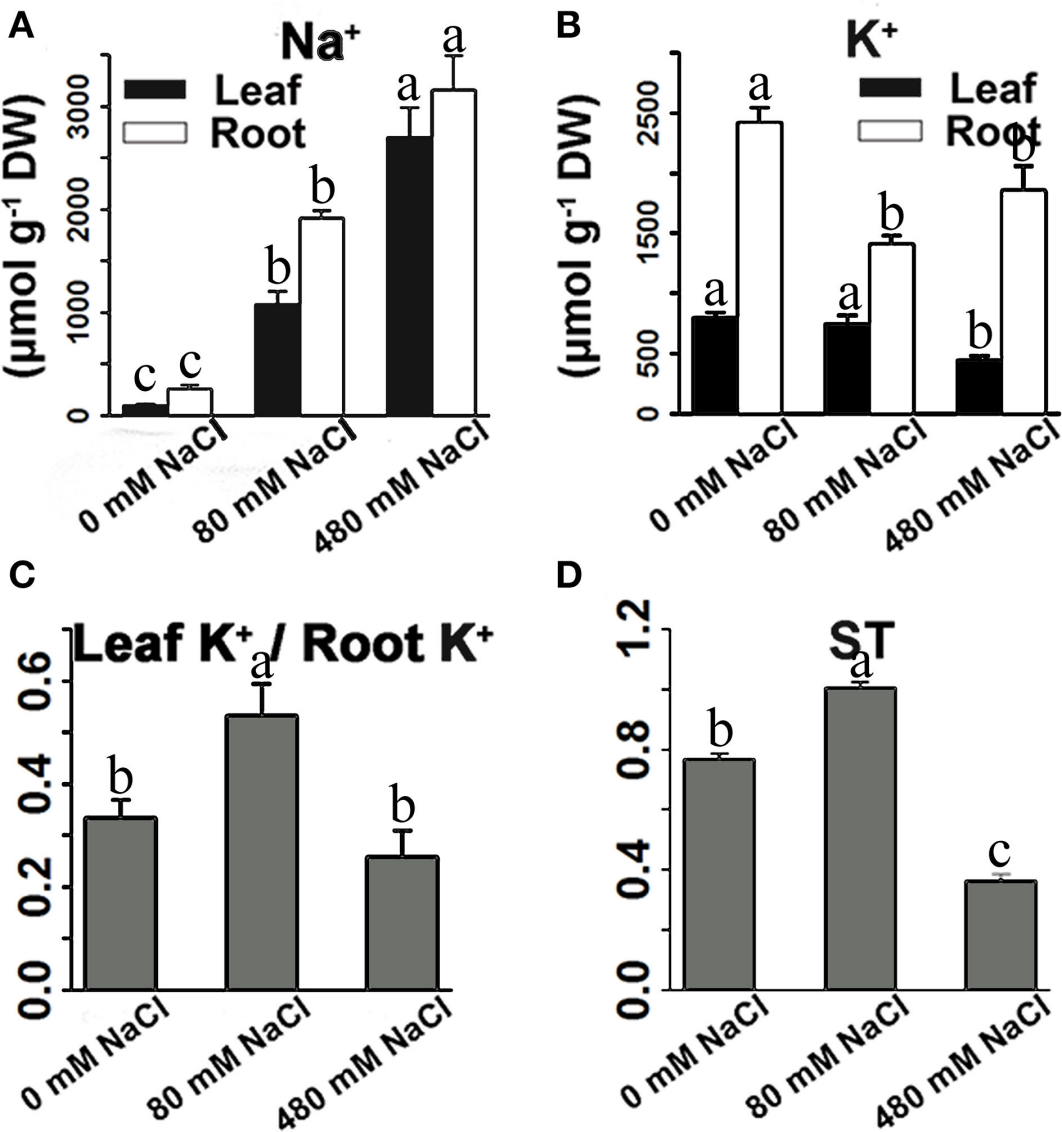
Effects of salinity stress on the Na^+^ content **(A)**, K^+^ content **(B)**, and ratio of leaf K^+^ to root K^+^ content **(C)**, and ST value **(D)** of *K. sieversiana*. The 30-d-old seedlings were subjected to 0, 80, and 480 mM NaCl for 30 days. Selective transport capacity of ion (ST) is calculated using following formula: (root Na^+^/K^+^)/(leaf Na^+^/K^+^). The greater ST value shows stronger capacity that root promotes K^+^ to transport to leaf. The values are means of four replicates (±SE). Different letters above bars indicate significant differences among treatments, according to a least significant difference test (*P* ≦ 0.05).

In roots, low-salinity stress influenced the accumulation of only Na^+^ rather than K^+^ (Figures 5A,B). The concentration of K^+^ in the roots was not reduced under low-salinity stress, but decreased under high-salinity conditions (Figure 5B, Figure S10). The leaf K^+^/root K^+^ ratio was enhanced only under low-salinity stress (Figure 5C). ST value is calculated using formula: (root Na^+^/K^+^) / (leaf Na^+^/K^+^). The greater ST value shows stronger capacity by which root promotes K^+^ transport to leaf. Indeed, low-salinity stressed plants showed greater ST value than control plants and high-salinity-stressed plants. We further analyzed the K^+^ transporter genes, and found that low-salinity stress did not influence the expression of any K^+^ transporter gene, but high-salinity stress strongly affected the expression of K^+^ transporter genes (Table 4). In roots, the expression of many K^+^ transporter genes was elevated by either low- or high-salinity stress. The expressions of some K^+^ transporter genes, including one *HAK* gene (TRINITY_DN235495_c0_g1|m.57939), three *AKT* genes (TRINITY_DN200663_c0_g1|m.36029, TRINITY_DN227311_c1_g1|m.32091, and TRINITY_DN230909_c0_g1|m.20114), and one *KAT* gene (TRINITY_DN219480_c0_g1|m.27505) were induced by only low-salinity stress. Furthermore, we annotated only four *HKT* genes that were considered fundamental salt-tolerant genes in glycophytic plants, and only one of these *HKT* genes was differentially expressed, being significantly reduced with increasing salinity. We also analyzed the data of the genes involved in ion compartmentation (Figures S5, S6, Table S5). Unfortunately, only one DEG was found in pathway of ion compartmentation and it's expression level was not high (Figures S5, S6, Table S5).

**Table 4:** Fold changes of differentially expressed K^+^ transporter genes under salinity stresses in the leaves and roots of *K. sieversiana*.

	**ID**	**80 mM NaCl/0 mM NaCl**		**480 mM NaCl/0 mM NaCl**		**Class (E-Value)**
		**Fold change**	***P*-value**	**Fold change**	***P*-value**	
Leaf	TRINITY_DN212979_c0_g1|m.24170	0.45	1	−1.71	0.00614303	HAK (2E-144)
	TRINITY_DN218493_c0_g1|m.31746	0.97	1	2.69	0.000198758	KAT (6E-9)
	TRINITY_DN231150_c1_g2|m.18958	−0.38	1	1.4	0.003415196	AKT (6E-11)
	TRINITY_DN226733_c2_g2|m.5637	0.33	1	1.04	0.005146315	KOR1 (2E-11)
Root	TRINITY_DN215699_c0_g1|m.11799	1.13	1.23E-05	1.13	0.013519435	HAK (0)
	TRINITY_DN235495_c0_g1|m.57939	3.96	0.008021979	0	1	HAK (1E-122)
	TRINITY_DN230084_c0_g16|m.36016	−1.08	0.132896845	−3.39	0.002005739	HKT (2E-60)
	TRINITY_DN200663_c0_g1|m.36029	0	NA	8.87	0.012071353	AKT (2E-6)
	TRINITY_DN204943_c0_g1|m.53965	−7.48	0.000936161	−7.55	0.008938157	AKT (3E-6)
	TRINITY_DN216976_c1_g1|m.51911	2.45	0.255148905	0	1	AKT (5E-8)
	TRINITY_DN227311_c1_g1|m.32091	−0.45	0.768778273	−2.78	0.004088713	AKT (6E-6)
	TRINITY_DN230909_c0_g1|m.20114	0.67	0.239123969	1.33	0.006113419	AKT (4E-12)
	TRINITY_DN219480_c0_g1|m.27505	0	NA	2.69	0.05653464	KAT (6E-163)
	TRINITY_DN227046_c1_g2|m.18771	−0.72	0.028601152	−1.8	2.60E-07	KOR1 (2E-76)
	TRINITY_DN227046_c2_g1|m.8396	−0.61	0.084303347	−1.85	2.94E-08	KOR1 (0)
	TRINITY_DN228656_c1_g2|m.8757	0.61	0.011664415	−1.75	3.16E-11	AKT (0)

*The 30-d-old seedlings were subjected to 0, 80, and 480 mM NaCl for 2 d*.

### Osmotic regulation

**Figure 6:**
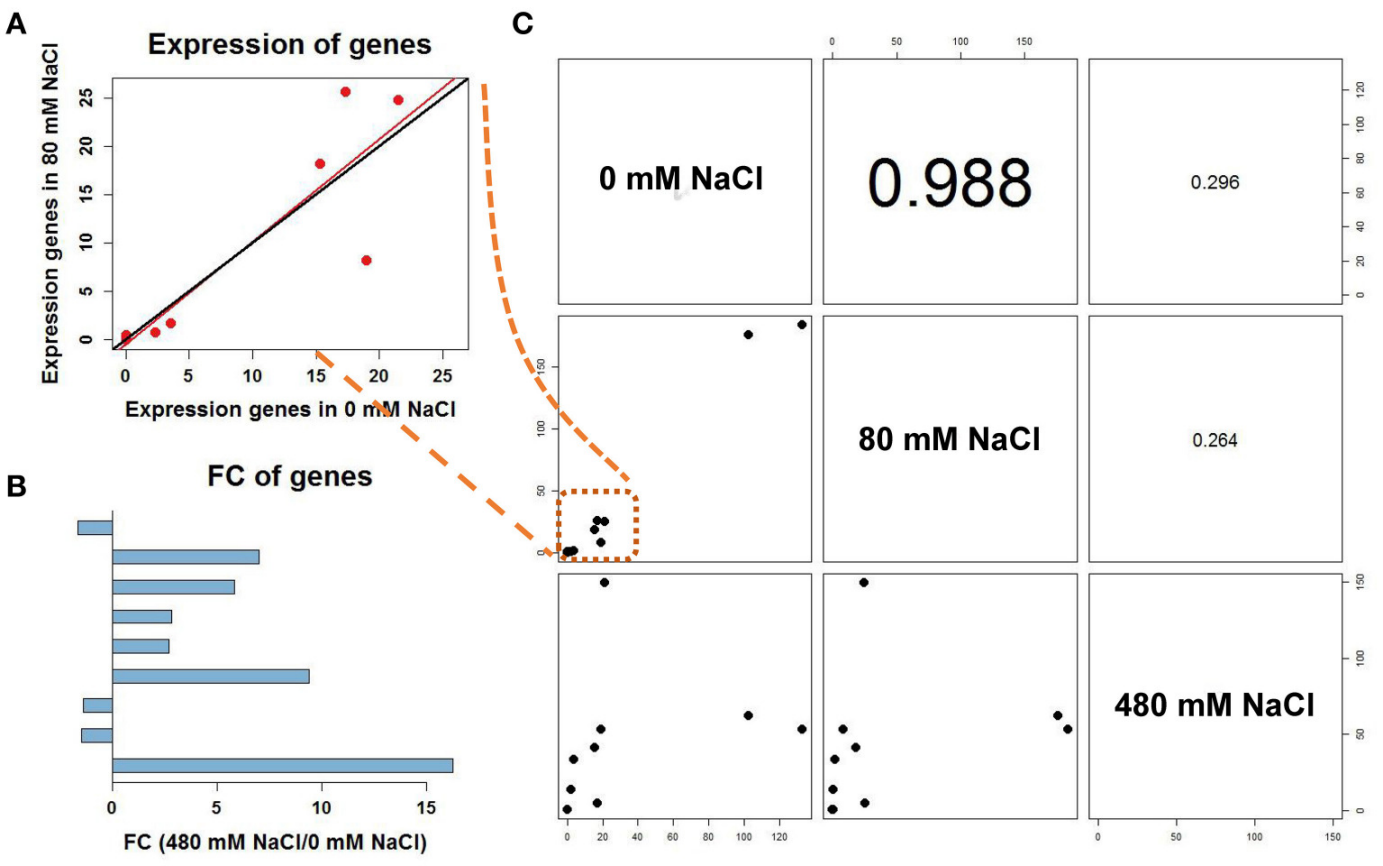
Correlation analyses among different treatments of differentially expressed unigenes involved in alanine, proline and sucrose metabolism. **(A)** The scatter plot of the seven genes in C was magnified. The fit line (red) between control and low salinity was close to the *y* = *x* line (black). **(B)** Fold change of differentially expressed genes under the 480-mM NaCl treatment to 0 mM NaCl. Up-regulated genes are shown to the right and down-regulated genes to the left. **(C)** Correlation coefficients for the expression levels of differentially expressed unigenes among different treatments.

We measured 17 components of low molecular weight carbohydrates and 20 amino acids in the leaves of *K. sieversiana* (Tables 5, 6). Amino acids play important roles in osmotic regulation in salinity stressed plants. Although some amino acids displayed an increasing tendency either under low-salinity stress or high-salinity stress, we were particularly concerned alanine because its concentration is highest in the leaves of *K. sieversiana*, is more than 50% of the total contents of the other 19 amino acids, and is far greater than that of proline. Alanine accumulation was induced under high-salinity stress but not under low-salinity stress. Of the 17 carbohydrates, the responses of sucrose and maltose to salinity stresses were noticeable. Low salinity decreased the maltose content but did not affect the accumulation of sucrose. However, high salinity can strongly increase the contents of these two carbohydrates (Table 6). In addition, low-salinity stress only produced small effects on the accumulations of erythrose and xylose, but high salinity decreased their accumulations. Glucose content was reduced under both stresses. High-salinity stress did not influence the accumulations of lactose and mannose, while low-salinity stress lowered their contents (Table 6). Based on the above biochemical results, alanine, proline, sucrose, and maltose may play important roles in the osmotic regulation of *K. sieversiana* under salinity stresses. To find possible molecular mechanisms, we analyzed the expression responses of their metabolism genes in the leaves and found nine DEGs. We conducted a correlation analysis for the expression levels of the genes among the three treatments (Figures 6A,C). We found that the fit line between control and low salinity was close to the *y* = *x* line, and the correlation coefficient was 0.988, indicating that low-salinity stress hardly influenced the expression levels of these genes. The correlation coefficients of the high-salinity treatment and low salinity and high-salinity treatment and control were 0.296 and 0.264, respectively. We further analyzed the fold changes of these genes caused by high-salinity stress and found that most genes were up-regulated, while only three genes were down-regulated (Figure 6B).

**Table 5:** Effects of salinity stress on the free amino acid contents in the leaves of *K. sieversiana*.

	**0 mM NaCl (μmol g^−1^ DW)**	**80 mM NaCl (μmol g^−1^ DW)**	**480 mM NaCl (μmol g^−1^ DW)**
Ala	6.93 ± 0.18^b^	8.20 ± 2.40^b^	11.33 ± 0.31^a^
Arg	1.47 ± 0.15^a^	1.68 ± 0.42^a^	1.88 ± 0.08^a^
Asn	2.12 ± 0.21^a^	2.47 ± 0.68^a^	3.11 ± 0.21^a^
Asp	0.27 ± 0.04^a^	0.40 ± 0.13^a^	0.33 ± 0.03^a^
Cys	0.00 ± 0.00^a^	0.00 ± 0.00^a^	0.00 ± 0.00^a^
Gln	2.86 ± 0.16^a^	1.55 ± 0.48^a^	3.47 ± 0.45^a^
Glu	0.18 ± 0.00^a^	0.27 ± 0.07^a^	0.23 ± 0.02^a^
Gly	0.59 ± 0.01^a^	0.83 ± 0.30^a^	0.88 ± 0.12^a^
His	0.22 ± 0.01^a^	0.28 ± 0.06^a^	0.27 ± 0.01^a^
Ile	0.88 ± 0.09^a^	1.02 ± 0.29^a^	1.00 ± 0.02^a^
Leu	1.02 ± 0.05^a^	1.05 ± 0.26^a^	1.21 ± 0.05^a^
Lys	2.62 ± 0.43^a^	2.05 ± 0.70^a^	2.53 ± 0.88^a^
Met	0.02 ± 0.00^a^	0.02 ± 0.00^a^	0.01 ± 0.00^b^
Phe	0.57 ± 0.01^a^	0.68 ± 0.21^a^	0.68 ± 0.01^a^
Pro	1.19 ± 0.07^a^	2.00 ± 0.62^a^	2.19 ± 0.24^a^
Ser	0.65 ± 0.02^a^	0.97 ± 0.32^a^	1.01 ± 0.03^a^
Thr	0.72 ± 0.03^a^	0.85 ± 0.25^a^	0.93 ± 0.00^a^
Trp	0.13 ± 0.01^a^	0.14 ± 0.04^a^	0.16 ± 0.01^a^
Tyr	0.26 ± 0.03^a^	0.38 ± 0.11^a^	0.48 ± 0.07^a^
Val	1.41 ± 0.08^a^	1.76 ± 0.51^a^	1.84 ± 0.04^a^

*The values are means ± SEs of four replicates. Different letters above the bars indicate significant differences among treatments according to a least significant difference test (P ≦ 0.05).The 30-d-old seedlings were subjected to 0, 80, and 480 mM NaCl for 30 d*.

**Table 6:** Effects of salt stress on the free carbohydrate contents in the leaves of *K. sieversiana*.

	**0 mM NaCl (μmol g^−1^ DW)**	**80 mM NaCl (μmol g^−1^ DW)**	**480 mM NaCl (μmol g^−1^ DW)**
Arabinose	0.93 ± 0.09^a^	0.68 ± 0.15^a^	0.63 ± 0.08^a^
Galactose	0.45 ± 0.07^a^	0.45 ± 0.09^a^	0.37 ± 0.12^a^
Erythrose	0.06 ± 0.00^a^	0.05 ± 0.00^a^	0.03 ± 0.01^b^
Mannose	1.63 ± 0.39^a^	0.62 ± 0.10^b^	0.89 ± 0.10^a^
Fructose	7.69 ± 0.79^a^	6.74 ± 1.24^a^	5.61 ± 0.49^a^
Trehalose	0.01 ± 0.00^a^	0.01 ± 0.00^a^	0.01 ± 0.00^a^
Xylose	0.62 ± 0.15^a^	0.45 ± 0.01^a^	0.24 ± 0.04^b^
Maltose	0.02 ± 0.01^b^	0.02 ± 0.00^c^	0.07 ± 0.01^a^
Raffinose	0.04 ± 0.01^a^	0.01 ± 0.00^b^	0.01 ± 0.00^c^
Glucose	40.85 ± 3.20^a^	25.04 ± 3.19^b^	18.08 ± 1.94^c^
Lactose	0.03 ± 0.00^a^	0.01 ± 0.00^b^	0.03 ± 0.00^a^
Pinitol	1.36 ± 0.11^a^	1.46 ± 0.17^a^	1.50 ± 0.23^a^
Sorbitol & Mannitol	0.03 ± 0.00^a^	0.02 ± 0.00^a^	0.02 ± 0.00^a^
Rhamnose	0.05 ± 0.00^a^	0.06 ± 0.00^a^	0.05 ± 0.01^a^
Stachyose tetrahydrate	0.01 ± 0.00^a^	0.00 ± 0.00^a^	0.00 ± 0.00^a^
Fucose	0.02 ± 0.00^a^	0.03 ± 0.01^a^	0.02 ± 0.00^a^
Sucrose	0.08 ± 0.03^b^	0.01 ± 0.00^b^	0.19 ± 0.02^a^

*The values are means ± SEs of four replicates. Different letters above the bars indicate significant differences among treatments according to a least significant difference test (P ≦ 0.05). The 30-d-old seedlings were subjected to 0, 80, and 480 mM NaCl for 30 d*.

## Discussion

### Growth, water balance, and oxidative stress

Many halophytes show much greater growth rates under moderate salinity conditions (50–400 mM NaCl) than those under non-salt conditions (Khan et al., 2000; Flowers and Colmer, 2008), and most halophytes can build dominant populations only under salinity stress conditions. The researches of extreme halophyte-specific property of salt tolerance may greatly improve our understanding of plant salt tolerance. Here, we investigated the salt tolerance mechanisms of the extreme halophyte *K. sieversiana*, which can survive in high salinity and high-pH environments. Figure 3 showed that *K. sieversiana* can survive and grow well under extreme conditions. We found that moderate salinity stimulated the growth and water uptake of *K. sieversiana* and that, even under 480-mM salinity conditions, *K. sieversiana* still maintained an extreme high WC (Figure 3, Table 3). The water balance of *K. sieversiana* under salinity stress is different from those of glycophytic plants in which the WC decreases with increasing salinity (Munns, 2002; Parida and Das, 2005; Munns and Tester, 2008). This is a specific adaptive strategy of *K. sieversiana* to deal with high salinity, because the metabolic activities involved in growth and development are highly dependent on water status of the cells or tissues. Transpiration involves an intense water flow traveling throughout the roots to leaves. The transport of water molecule from root to leaf mostly occurs through specialized dead vessels (xylem); however, in some specific metabolic processes or under some extreme stress conditions, water molecules need to be delivered through living cells (Maurel et al., 2015). The transport process of water molecules through living cells may be accurately controlled and regulated (Maurel et al., 2015), which is dependent on AQPs. Here, extreme high salinity could still enhance the WC of *K. sieversiana*. The increasing WC may not be related to transpiration because high salinity usually decreases transpiration. Because leaves of *K. sieversiana* are succulent and small, it is difficult to accurately measure the transpiration. However, although we did not record transpiration data, we believe that the high salinity may have reduced transpiration. We further investigated the mechanisms underlying the high WC using the RNAseq analysis and morphological data. The leaves showed increasing succulence (Figure 3D, Table 3). The RNAseq results indicated that low-salinity stress up-regulated 10 of 12 *AQP* DEGs in the roots, while high salinity only up-regulated 2 of 12 *AQP* DEGs in the roots (Figure 4). Higher expression levels of the two *AQP* genes (TRINITY_DN217586_c2_g1|m.14771 and TRINITY_DN219547_c1_g1|m.22582) under high-salinity stress may facilitate the water uptake of *K. sieversiana* and contribute to its strong salinity and osmotic stress tolerances. Most *AQP* genes were significantly up-regulated in low-but not high-salinity stressed roots, which may partly explain why *K. sieversiana* grows better under low-salinity than under control condition.

Salinity stress usually induces the formation of reactive oxygen (ROS). These reactive oxygens may destroy lipids, protein, and nucleic acids, and disrupt normal metabolism. Higher plants possess antioxidative enzyme system that can clean reactive oxygen and against oxidative stress. Expression of antioxidative enzyme genes are elevated under salinity stress (Parida and Das, 2005). Although we identified a number of the antioxidative enzyme genes including 20 *Cu/Zn-SOD* genes, 13 *CAT* genes, 27 *Mn-SOD* genes, 30 *GST* genes, eight *GPX* genes, and 135 *peroxidaxe* genes, only relatively fewer genes were up-regulated (Figure S9). Most antioxidative enzyme genes might redundently function in salinity tolerance, because the up-regulated antioxidative enzyme genes may be more important in salinity stress response of *K. sieversiana*.

### Ion balance

Na^+^ uptake usually competes with K^+^ uptake because K^+^ transporters have difficulty discriminating between K^+^ and Na^+^ (Zhu, 2003; Parida and Das, 2005). Competition between K^+^ and Na^+^ for passing or binding transporters may occur anywhere, such as, the root-soil interface and within the roots. A high concentration of intracellular Na^+^ may limit the activities of cytosolic enzymes and affect membrane stability (Zhu, 2003; Parida and Das, 2005). The ability and mechanism to tolerate Na^+^ stress might differ between species of plants. For most glycophytic plants, the main Na^+^ tolerance mechanisms are Na^+^ exclusion from the shoot to the root and the partitioning of the accumulated Na^+^ among organs and within cells (Plett and Møller, 2010). For example, in wheat, rice, tomato, and Arabidopsis, some K^+^/Na^+^ transporters mediate Na^+^ exclusion from the shoot to the root or from the root to rhizosphere with the goal of decreasing Na^+^ accumulation in above ground organs (Parida and Das, 2005; Munns and Tester, 2008; Olías et al., 2009). A *SOS1* gene from extreme halophyte *Salicornia brachiata* enhances Na^+^ loading in xylem and confers salt tolerance of transgenic tobacco plants (Yadav et al., 2012). Thus, lower Na^+^ contents in above ground parts were perceived to be important indicators of salt tolerance for glycophytic plants. However, for some extreme halophytes, salt components can make up to 50–60% of the dry weight (Khan et al., 2000), and refusing Na^+^ may not be an indispensable Na^+^ tolerance mechanism for these plants. Similarly, *K. sieversiana* can accumulate higher concentrations of Na^+^ in shoots under salinity stresses, even under low salinity stress (80 mM) (Figure 5), suggesting that salinity stress may urge the accumulation of Na^+^ in the above ground organs of *K. sieversiana*. Compared with glycophytic plants, under a similar low-salinity condition, *K. sieversiana* accumulated a much higher concentration (~1,100 μmol/g) of Na^+^ in leaves, which failed to threaten its growth. Such high concentrations of Na^+^ may severely influence some glycophytic plants, even killing them (Chen W. C. et al., 2009; Chen W. et al., 2009; Yang et al., 2014).

We surveyed the expression levels of typical genes, such as, *HKT* genes, *NHX* genes, *PP-ATPase* genes, *V-ATPase* genes, and the SOS system involved in Na^+^ tolerance in the roots (Figure 2, Figures S5, S6 and Table 4, Table S5). The expression of some K^+^ transporter genes was increased by only the high-salinity stress, while the expression of other K^+^ transporter genes was induced by only the low-salinity stress (Table 4). This indicated that the regulation of gene expression in *K. sieversiana* was salinity-dependent. Although we did not identify the electrophysiological features of these transporters, we propose that the increased expression of the K^+^ transporter genes may facilitate the Na^+^ uptake of *K. sieversiana* under salinity stresses. In addition, up-regulations of the K^+^ transporter genes may facilitate *K. sieversiana* to compartmentalize Na+ into vacuole because these K^+^ transporters may locate on vacuole membrane. Higher concentrations of Na^+^ should increase *K. sieversiana*'s ability to cope with the strong osmotic stress caused by higher salinity or extreme dry weather. In addition, we only identified four *HKT* genes in *K. sieversiana* and did not find that their expression levels were up-regulated, which was not consistent with some glycophytic plants, such as, wheat and rice, in which *HKT* genes expression are enhanced to increase the rate of Na^+^ exclusion from shoots into roots or from roots into the rhizosphere (Ren et al., 2005; Yang et al., 2014). The expression of a *SOS1* gene in the roots was up-regulated only by low-salinity stress. Glycophytic plants compartmentalize massive amount of Na^+^ into vacuole by NHX, PP-ATPase, and V-ATPase (Zhu, 2003; Parida and Das, 2005). Surprisingly, neither low-salinity nor high-salinity enhanced the expression of these genes in *K. sieversiana* (Figures S5, S6, Table S5). Thus, some typical salinity-tolerant genes identified in glycophytic plants may have different roles or do not function in *K. sieversiana*.

### Osmotic regulation

Plants generally compartmentalize Na^+^ into vacuoles to avoid Na^+^ toxicity in the cytoplasm. To combat the osmotic stress caused by higher concentrations of Na^+^ in the vacuoles, plants also accumulate compatible solutes in the cytoplasm, such as, betaine, proline, free sugar, and polyalcohol (Parida and Das, 2005; Munns and Tester, 2008). Proline has been highly studied and was perceived as the most important osmotic solute in glycophytic plants (Hasegawa et al., 2000; Munns, 2002; Flowers and Colmer, 2008; Munns and Tester, 2008); however, our results showed that high salinity dramatically enhanced the alanine content of *K. sieversiana*, which was much greater than the proline content. This revealed that alanine may have greater importance in the osmotic regulation of *K. sieversiana* than proline. We suggest that alanine may play important roles in plant salt tolerance and should be investigated in the future.

Low molecular weight carbohydrates are not only substrates of carbon and energy metabolisms but also have multiple roles in plant growth, development, and stress responses (Gupta and Kaur, 2005). These carbohydrate molecules could be signaling molecules or osmotic regulators, and carbon materials are stored for building stress-response proteins and compounds (Gupta and Kaur, 2005). Many stress conditions can strongly influence the carbohydrate metabolism of plants. A lot of stress-responsive genes can be induced by carbohydrate treatments (Gupta and Kaur, 2005). Thus, carbohydrate metabolism and stress tolerance may have a complicated interaction. In this study, we found that salinity stress may strongly affect the carbohydrate metabolism of *K. sieversiana*. The accumulation of many carbohydrate components was inhibited by either low salinity or high salinity. Low salinity decreased the sucrose content but did not affect the accumulation of maltose; however, high salinity strongly increased the contents of the two carbohydrates (Table 6). *K. sieversiana* survived a long time under high-salinity conditions and may have evolved complicated physiological and biochemical mechanisms for resisting ion injury and osmotic stress. Changes in the solute carbohydrate accumulation may be an adaptive strategy of *K. sieversiana*. Solute carbohydrates may play different roles at different salinity levels. Maltose and sucrose may play important roles in the osmotic regulation of *K. sieversiana* under high-salinity conditions but not under low-salinity stress. This may depend on the intensity of the osmotic stress or ion injury and whether the carbohydrate metabolism was altered or signaling was triggered. We analyzed the expressions of 10 genes related to the synthesis and metabolism of sucrose, alanine, and proline. We found that low-salinity and control treatments have similar gene expression patterns with a 0.988 correlation coefficient (Figure 6). In contrast, these two treatments have small correlation coefficients with the high-salinity treatment (Figure 6). This indicated that *K. sieversiana* employed diverse osmotic regulatory mechanisms under different salinity levels.

**Figure 7:**
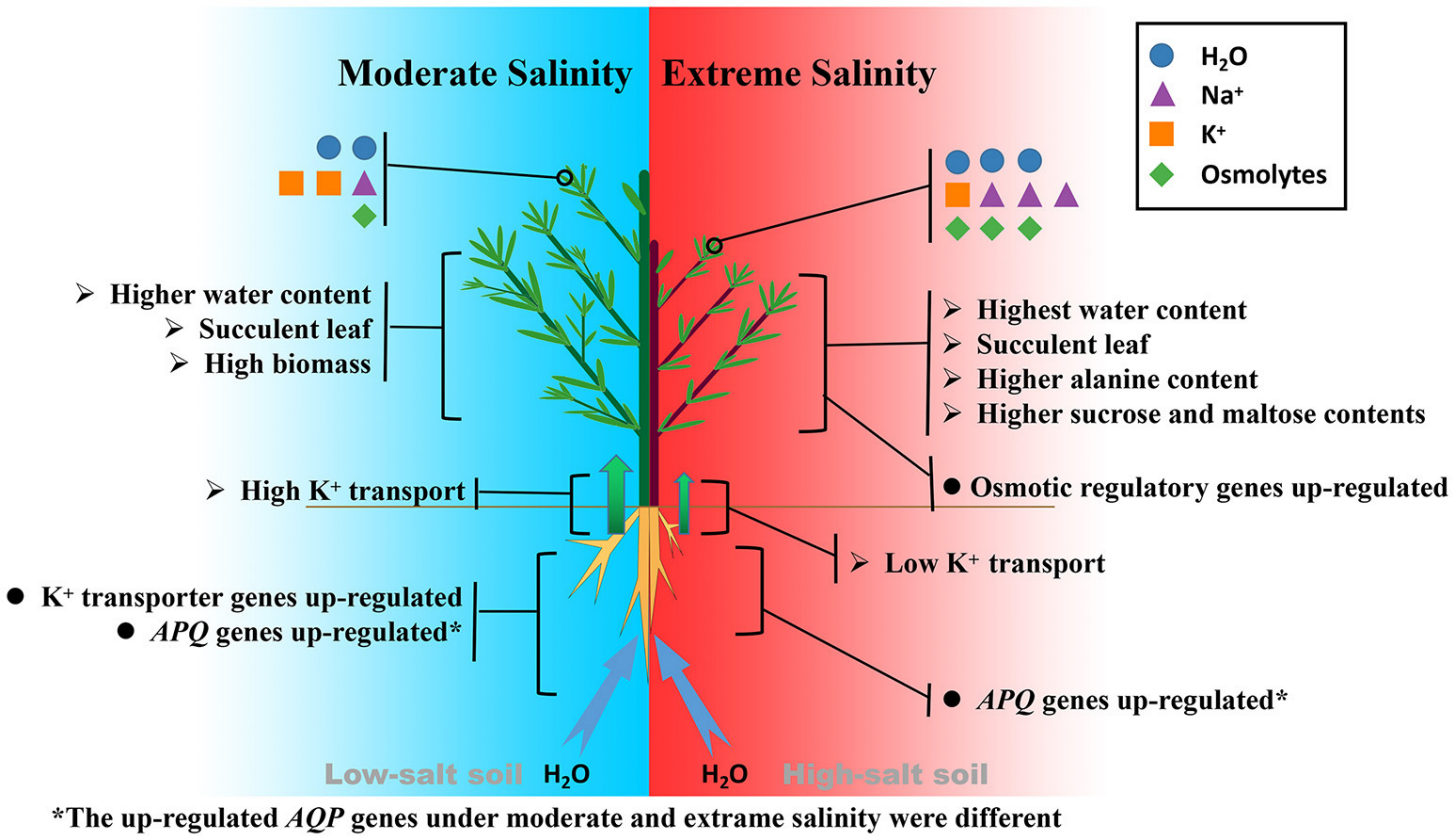
Comparison of salt tolerance mechanisms of *K. sieversiana* under moderate and extreme salinity stresses.

Thus, the biochemical analysis revealed that, under salinity stresses, *K. sieversiana* increased succulence and promoted the accumulation of Na^+^, alanine, sucrose, and maltose in shoots to regulate the osmotic potential (Figure 7). The greater accumulations of alanine, sucrose, and maltose were observed under high- not under low-salinity stress. This suggested that different osmotic regulatory mechanisms were employed under low- and high-salinity stresses, which was supported by the correlation analyses of genes related to the metabolisms of sucrose, alanine, and proline (Figures 6, 7). DEG data showed that some *AQP* and K^+^ transporter genes may be important for water uptake and ion balance, respectively, but different members of the same families were differentially induced under low- and high-salinity stresses. Many *AQP* genes were significantly up-regulated in low- but not high-salinity stressed roots, and these highly expressed *AQP* genes may allow low-salinity stressed *K. sieversiana* plants to uptake more water than control plants. In addition, it was speculated that low salinity promoted K^+^ transport from the roots to the shoots based on multiple lines of evidence. Firstly, the ratio of K^+^ to root K^+^ increased under low- but not high-salinity stress. Secondly, K^+^ content of root was higher in high-salinity stress than that in low-salinity stress. This demonstrated that increasing Na^+^ concentration surrounding the roots did not decrease the accumulation of K^+^ in the roots but enhanced it. These data indicated that the increasing K^+^ content in high-salinity stressed roots may be attributable to increasing osmotic stress not to increasing Na^+^ toxicity because high concentrations of Na^+^ surrounding the roots usually inhibited K^+^ uptake. Furthermore, compared with control treatment, low-salinity stress decreased the accumulation of K^+^ in roots, which may be a regulative process that mediates transport of K^+^ from the roots to the shoots. Finally, ST value, as an indicator of the capacity that root promotes K^+^ transport to leaf, was much greater in low-salinity stressed plants than those in control plants and high-salinity stressed plants. Although multiple evidences make us to speculate that low salinity urged K^+^ transport from the roots to the shoots, more physiological, and molecular evidences may be needed to exhaustively figure out this issue. Lower K^+^ content of low-salinity stressed root also might be attributable to its greater growth rate and higher water content. Taken together, we propose that low salinity might facilitate *K. sieversiana* to uptake more water and transport more K^+^ from roots to shoots, resulting in the better growth of *K. sieversiana* compared with control plants (Figure 7).

## Data availability statement

All raw data files are available from the SRA database (SRP122261: PRJNA415916).

## Author contributions

Experiment designed: CY and LZ. Data analysis: CY, LZ, and FS; Physiological Experiment: ZY, QG, SL, and SM. Molecular Experiment: CY, LZ, and HW. Manuscript writing: CY and LZ.

### Conflict of interest statement

The authors declare that the research was conducted in the absence of any commercial or financial relationships that could be construed as a potential conflict of interest.
