# Supplementary material for: Transcriptomic Profiling and Physiological Responses of Halophyte Kochia sieversiana Provide Insights into Salt Tolerance
Source: Front Plant Sci. 2017 Nov 24;8:1985. doi: 10.3389/fpls.2017.01985 (PMC5705942; doi:10.3389/fpls.2017.01985)
Supplement: Supplementary file 1 [file FiguresandTables.pdf]

Supplementary Material

Transcriptomic profiling and physiological responses of halophyte *Kochia sieversiana* provide insights into salt tolerance

Long Zhao[1], Zongze Yang[1], Jiaopin Guo[1], Shun Maoa[1], Shaoqiang Li[1], Fasheng Sun[1], Huan Wang[2] and Chunwu Yang[1*]

[1]Key laboratory of Molecular Epigenetics of Ministry of Education (MOE), Northeast Normal University, Changchun 130024, China

[2]Department of Agronomy, Jilin Agricultural University, Changchun 130018, China

[*]**Correspondence:**

Dr. Chunwu Yang

E-mail: 45447087@qq.com

Supplementary Figures and Tables

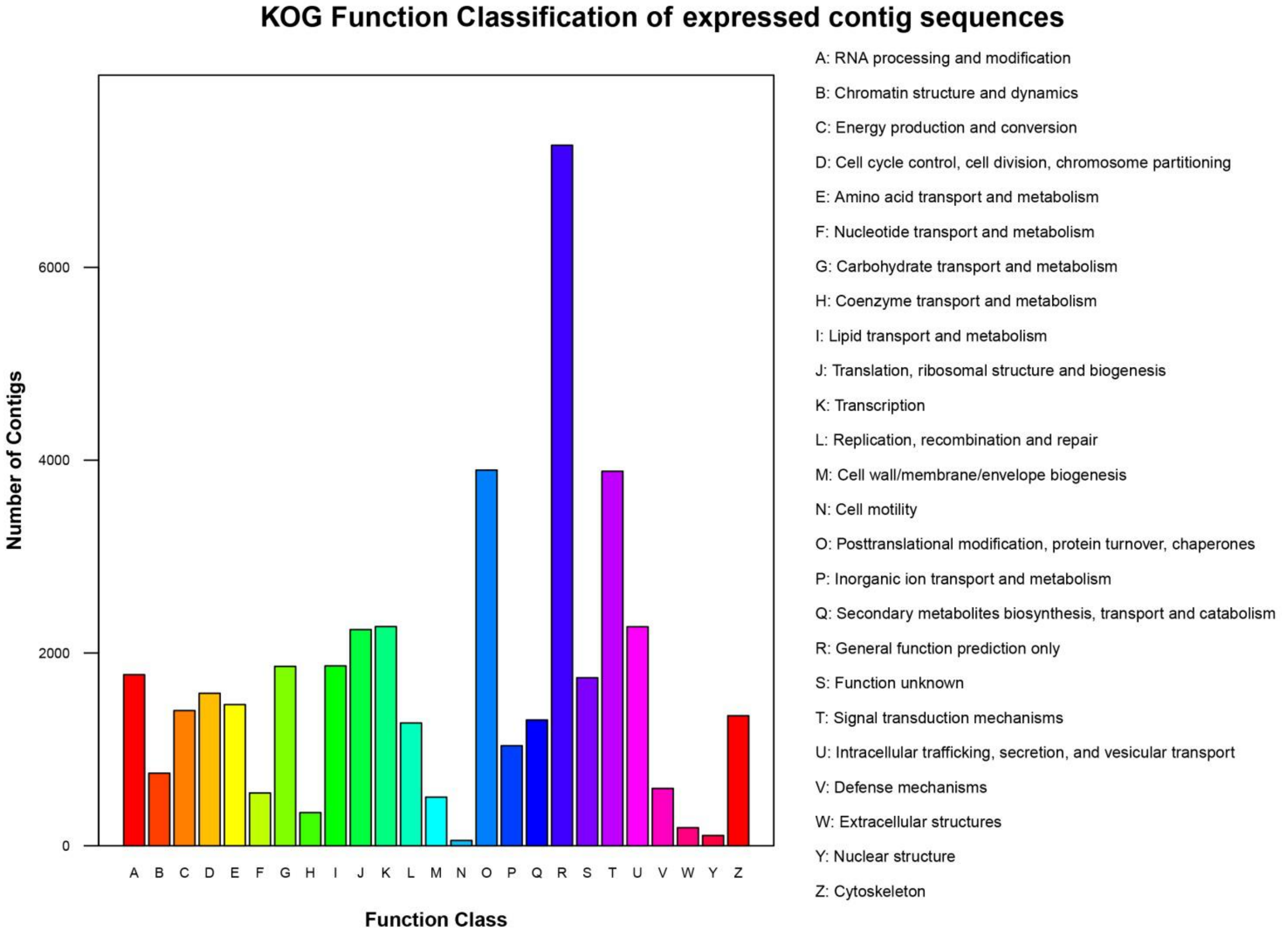

Supplementary Figure 1.COG functional classification of expressed contig sequences of *K. sieversiana*. The 30-d-old seedlings were subjected to 0, 80, and 480mM NaCl for 2 d.

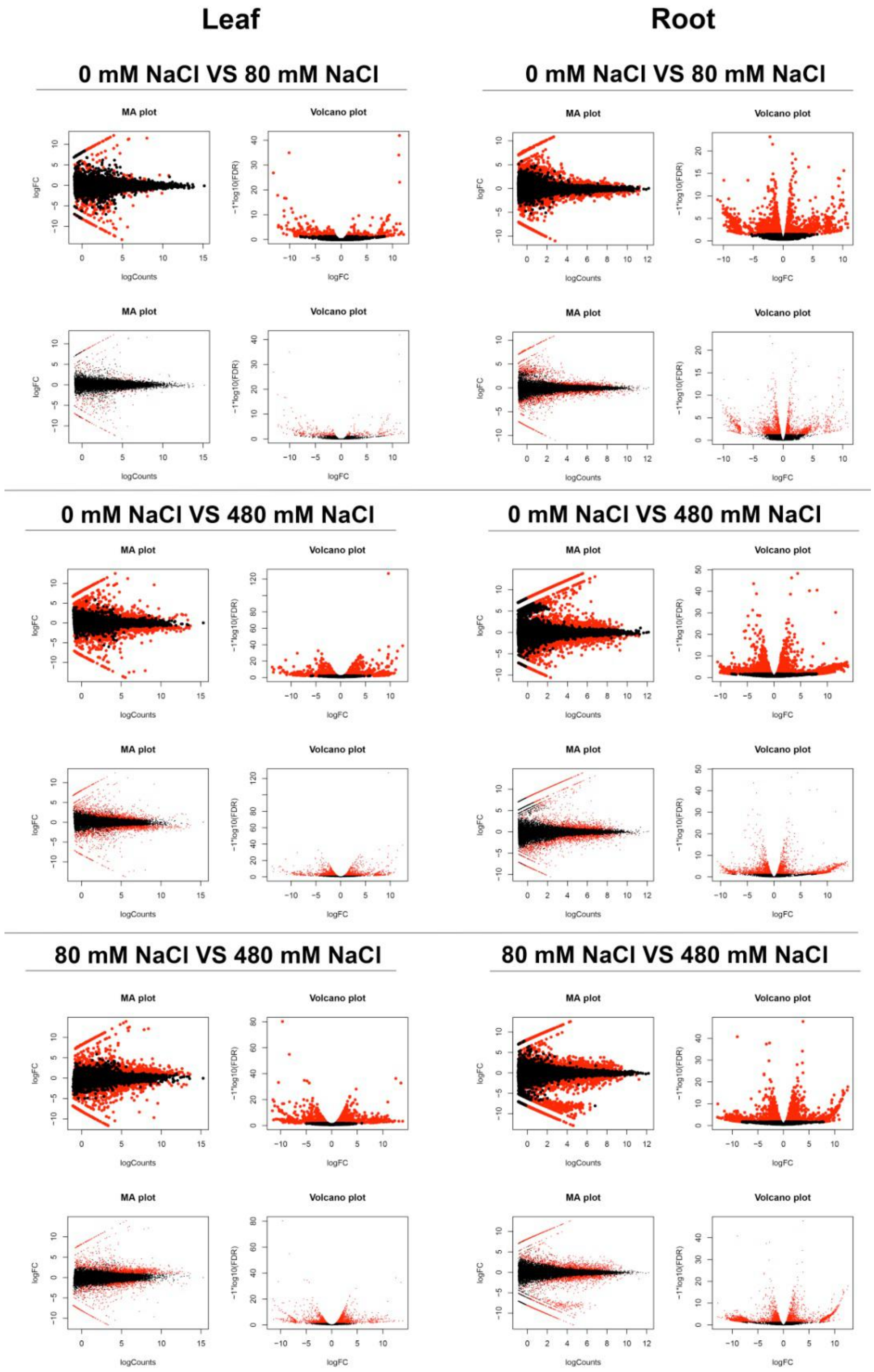

Supplementary Figure 2. MA and volcano plotsof responses to different NaCl treatments in the leaf and root of *K. sieversiana*. LogFC represents the log2(Fold change), logCounts represents the log2(number of counts),and −1*log10(FDR) represents $-1\times\log_{10}$(false discovery rate). The 30-d-old seedlings were subjected to 0,80 and 480mM NaCl for 2 d.

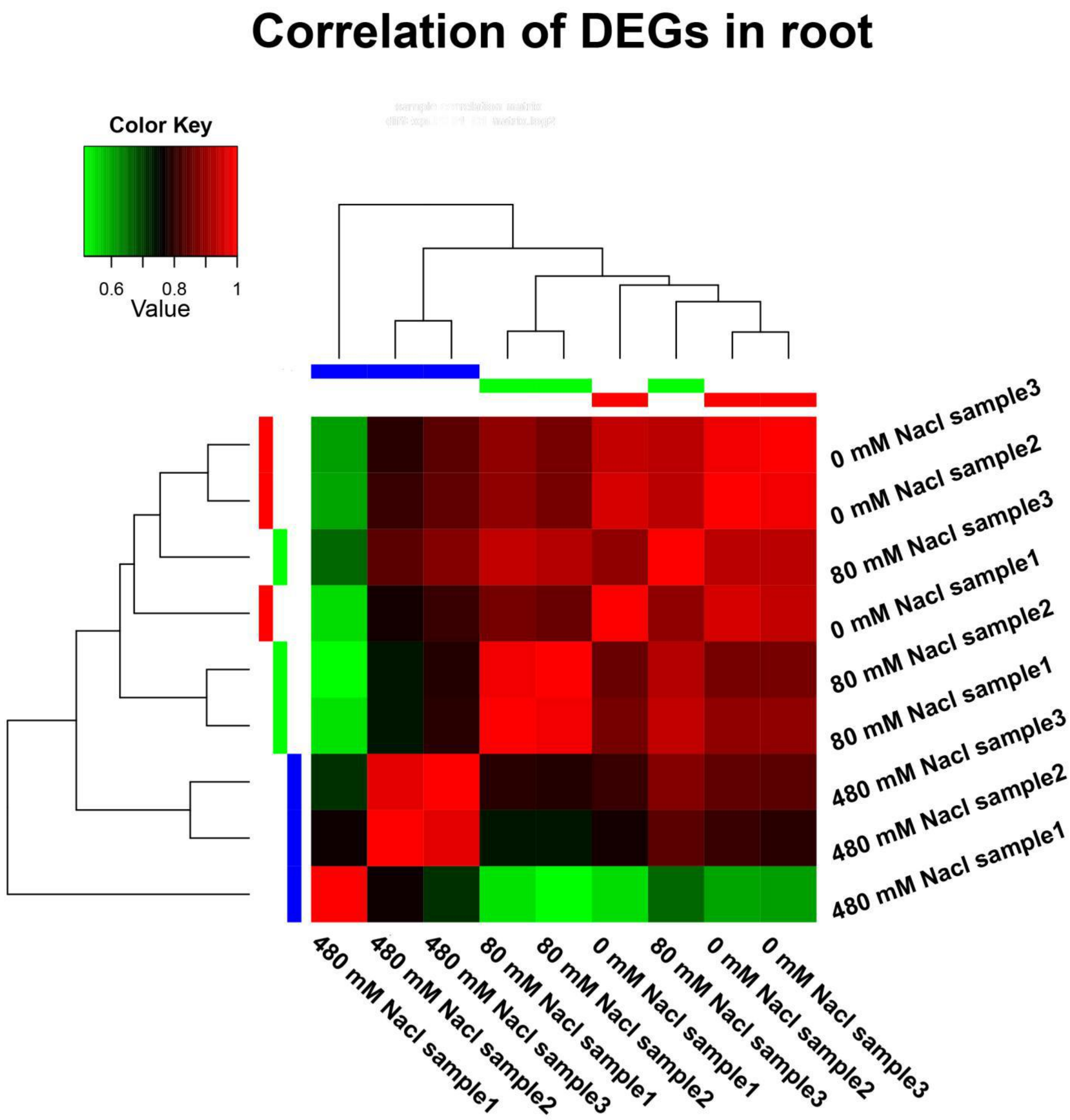

Supplementary Figure 3. Correlations of gene expression levels of nine root samples. The 30-d-old seedlings were subjected to 0, 80, and 480mM NaCl for 2 d.

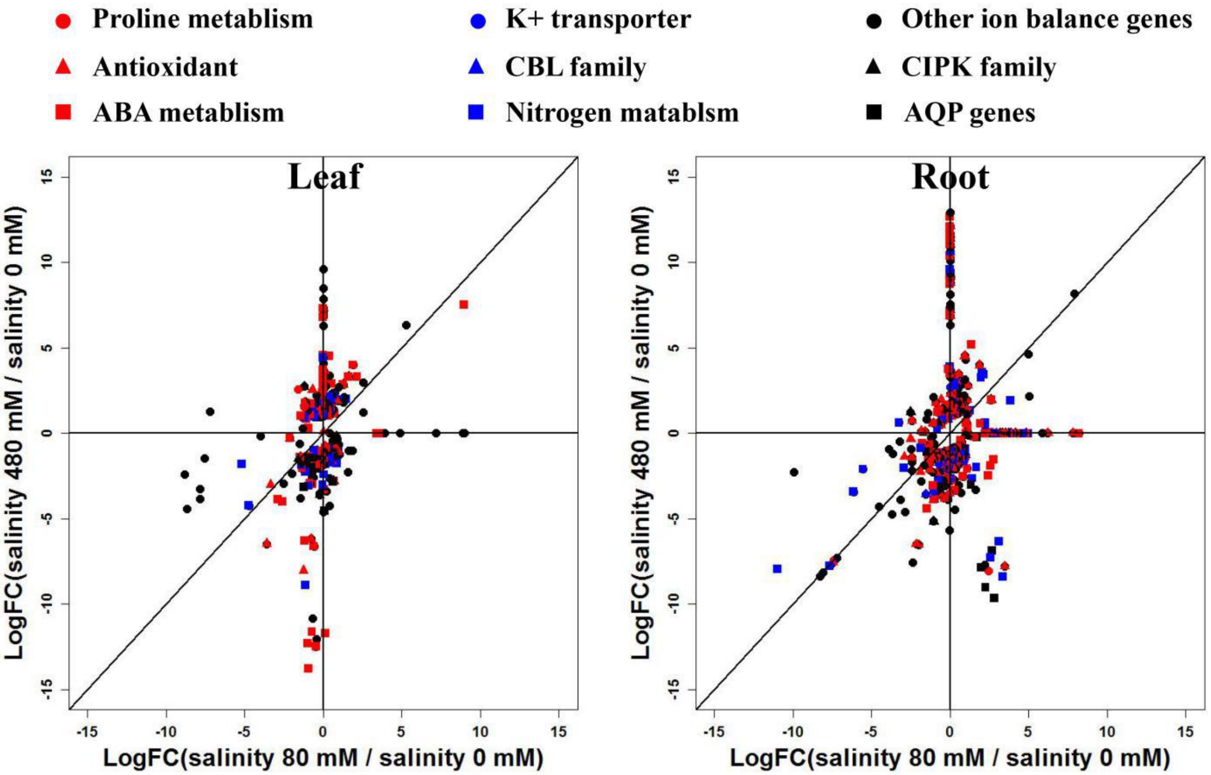

Supplementary Figure 4. Effects of different salinity levels on the expression of salinity-tolerant unigenes in the leaves and roots of *K. sieversiana*. Icons represent differentially expressed salinity tolerance genes. *X*-axis= Log2 of the ratio of 80-mM salinity to control in gene expression level, and y-axis = Log2 of the ratio of 480-mM salinity to control. The icons close to the *x*-axis indicated unigenes that have small expression differences between 80mM salinity and control, the icons close to the *y*-axis indicate unigenes that have small expression differences between 480mM salinity and control, and the icons that fell on the diagonal *x*=*y* line indicated that 80 and 480mM salinity produced identical effects on the expression of the unigenes. The 30-d-old seedlings were subjected to 0, 80, and 480-mM NaCl for 2 d.

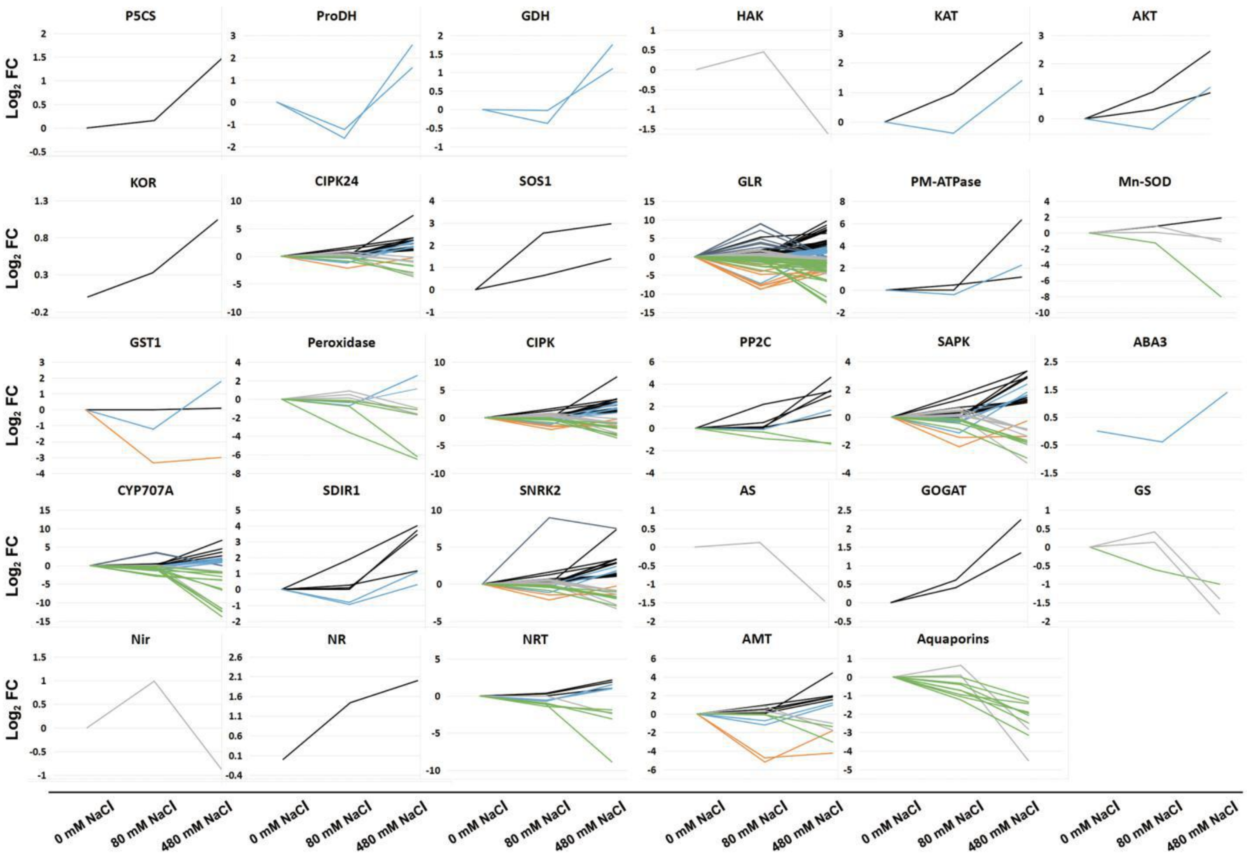

Supplementary Figure 5. Effects of salinity stress on the expression of different members of salinity-tolerant gene families in leaves of *K. sieversiana*. Each curve represents a member of a certain gene family. The 30-d-old seedlings were subjected to 0, 80, and 480mM NaCl for 2 d.

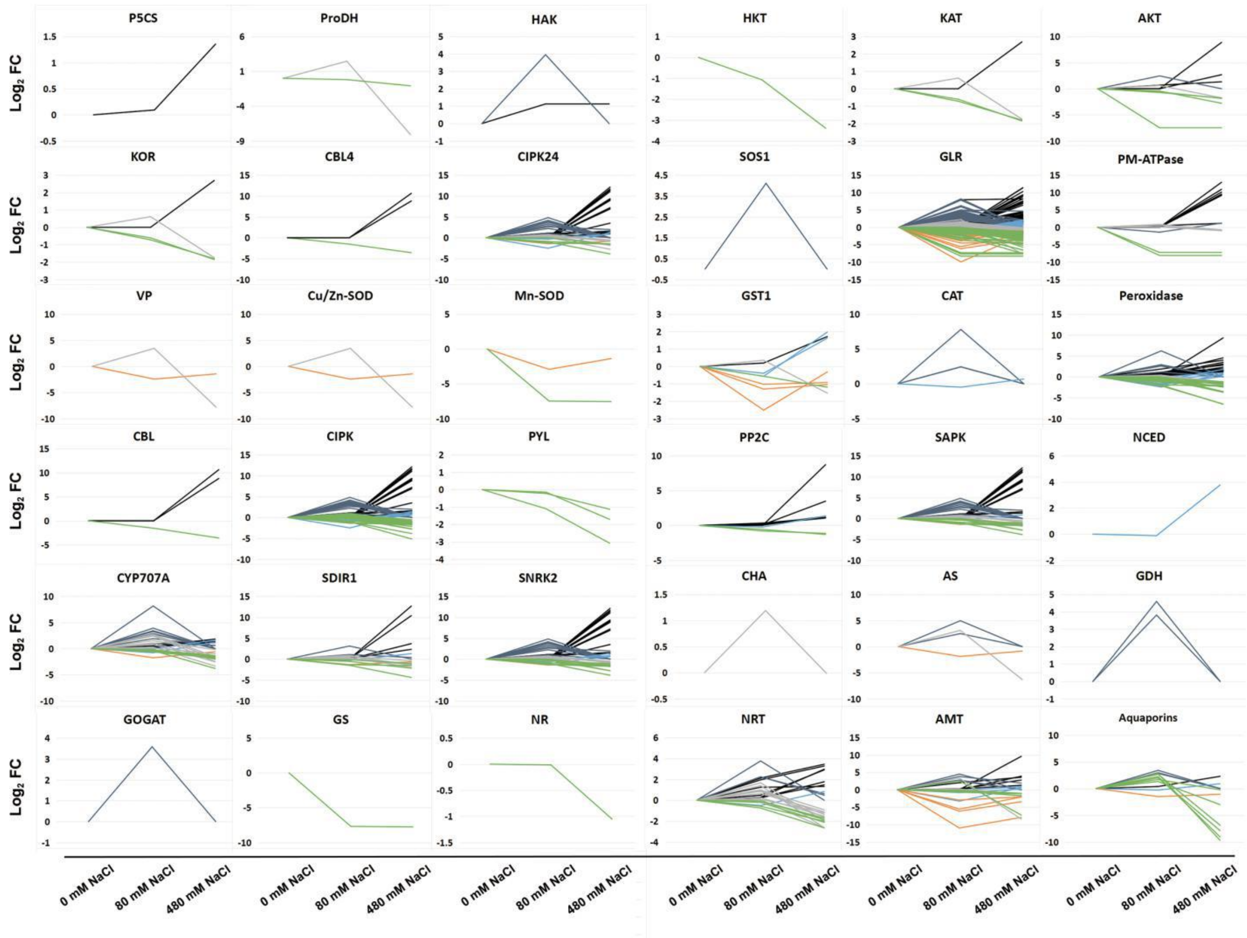

Supplementary Figure 6. Effects of salinity stress on the expression levels of different members of salinity-tolerant gene families in roots of *K. sieversiana*. Each curve represents a member of a certain gene family. The 30-d-old seedlings were subjected to 0, 80 and 480mM NaCl for 2 d.

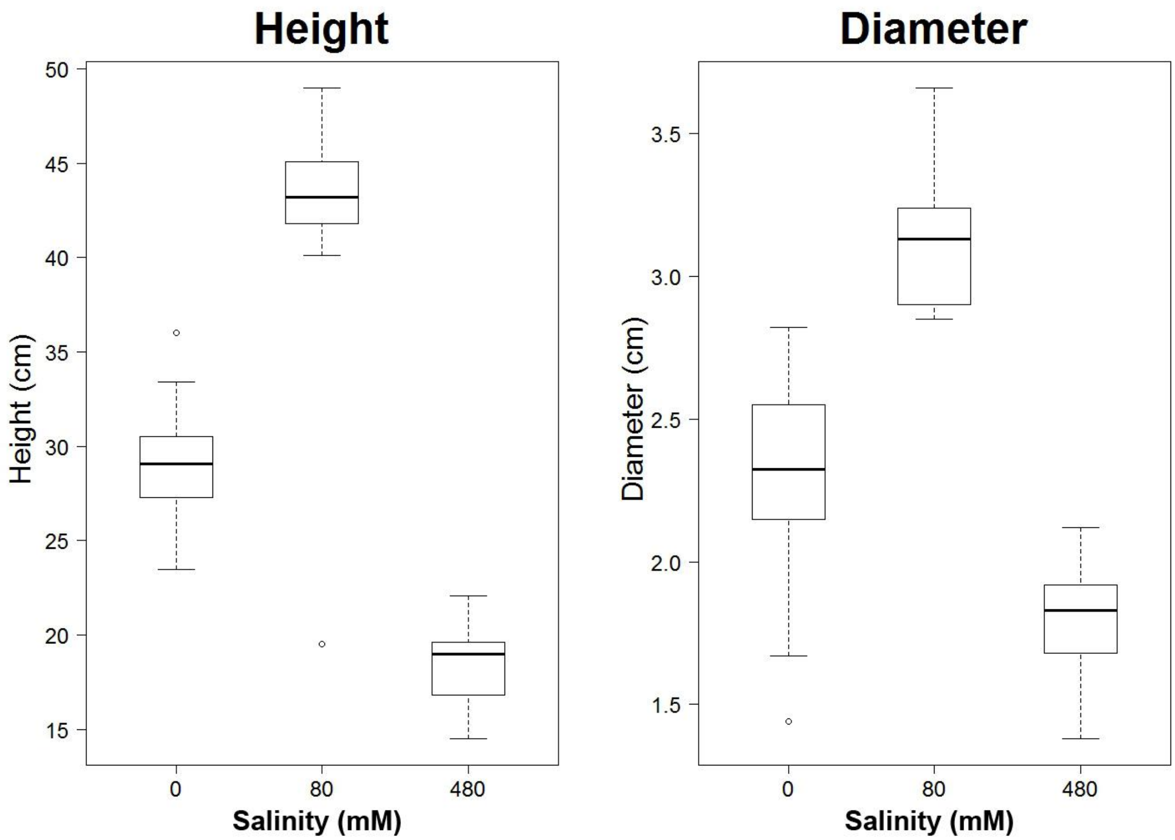

Supplementary Figure 7. Effects of salinity stress on the height (A) and the stem diameter (B) of *K. sieversiana*. Thirty individuals for each treatment were used in the Boxplot. The 30-d-old seedlings were subjected to 0, 80, and 480mM NaCl for 30 d.

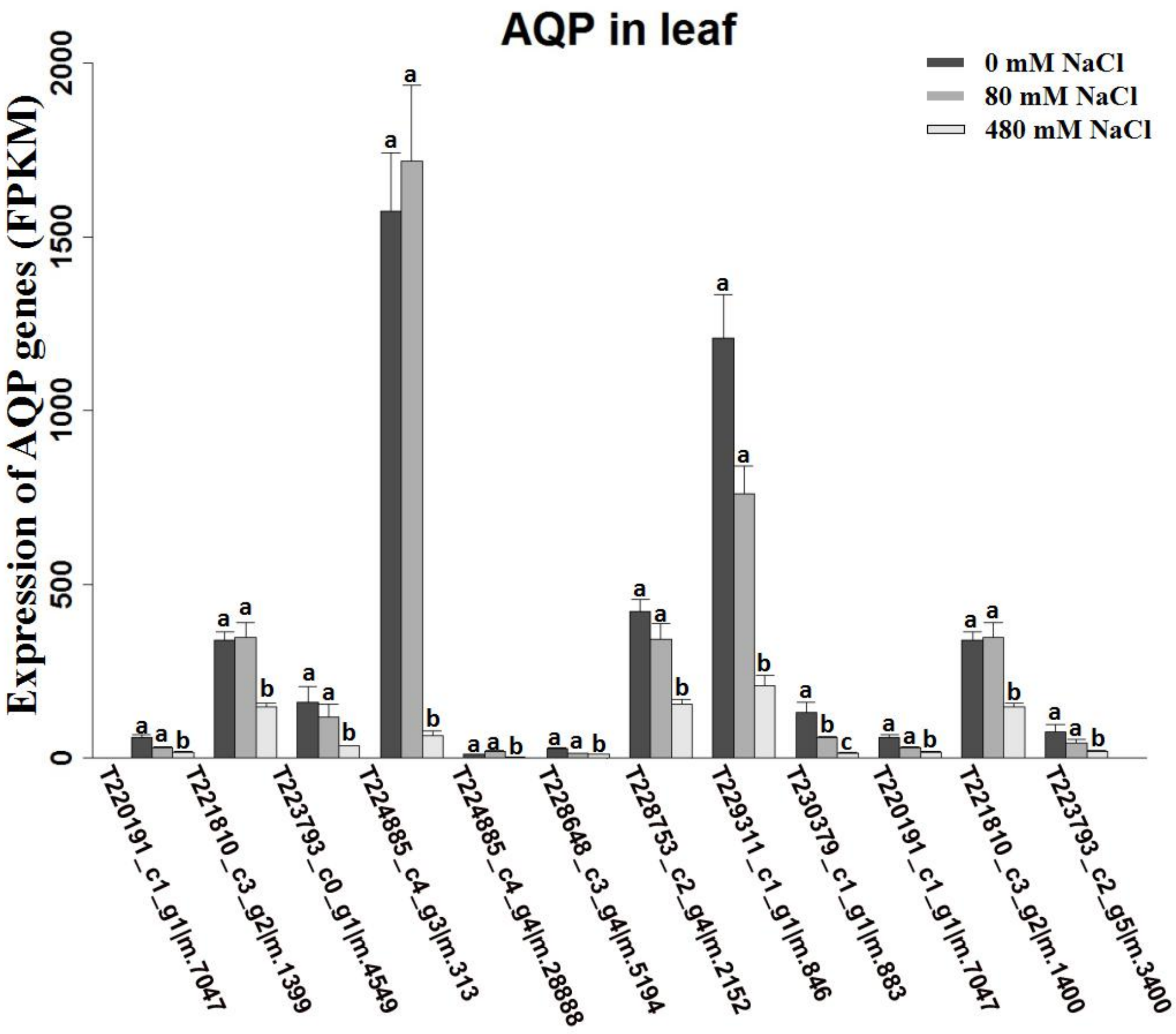

Supplementary Figure 8. Expression levels of aquaporin-like unigenes in leaves of *K. sieversiana* under different salinity treatments. The values are means of three replicates (± SE). The 30-d-old seedlings were subjected to 0,80, and 480mM NaCl for 2 d. Different letters above the bars indicate significant differences among treatments, according to a least significant difference test (P ≦ 0.05).

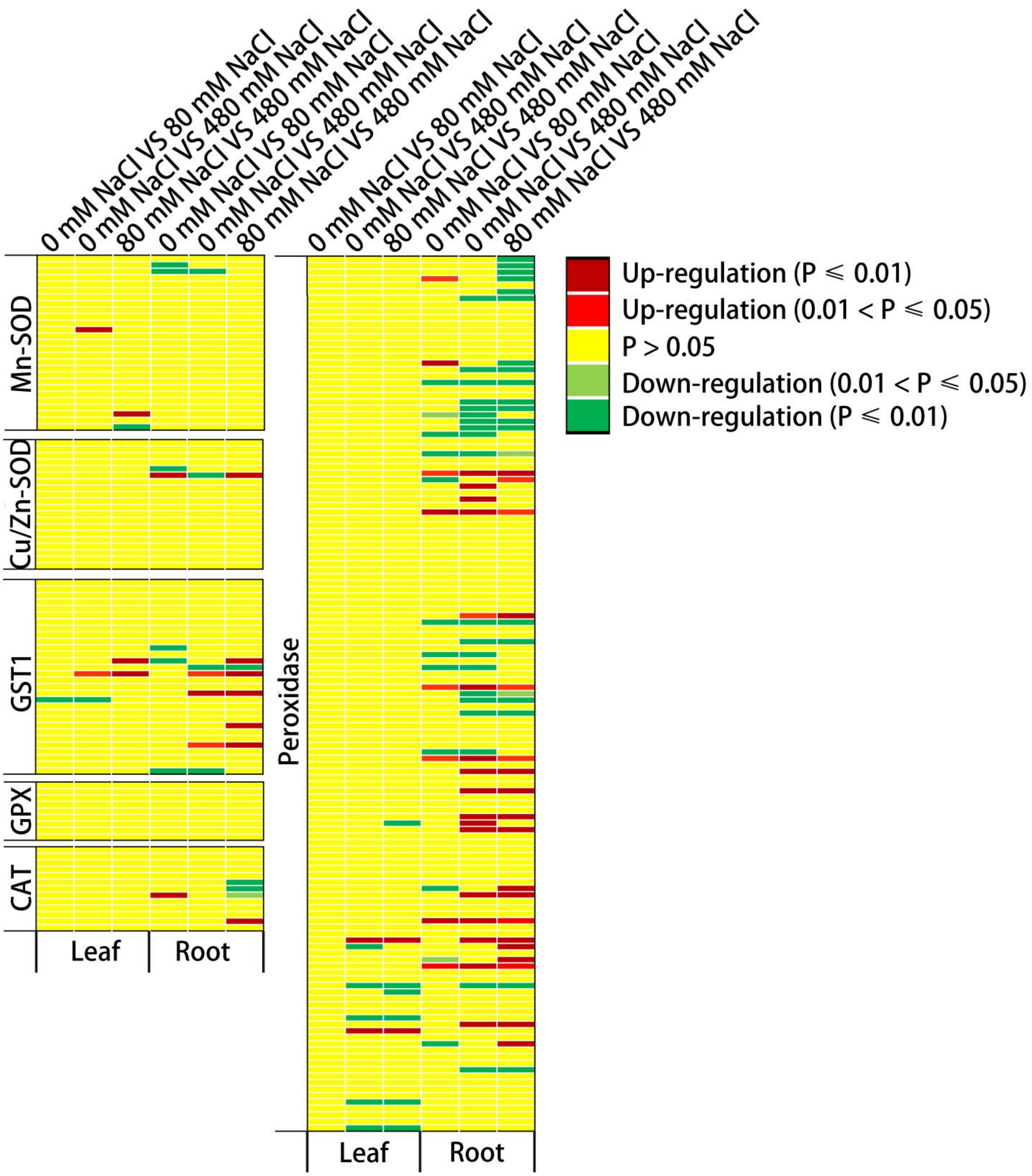

Supplementary Figure 9. Effects of salinity stresses on expression of antioxidative enzyme genes. The 30-d-old seedlings were subjected to 0, 80 and 480mM NaCl for 2 d.

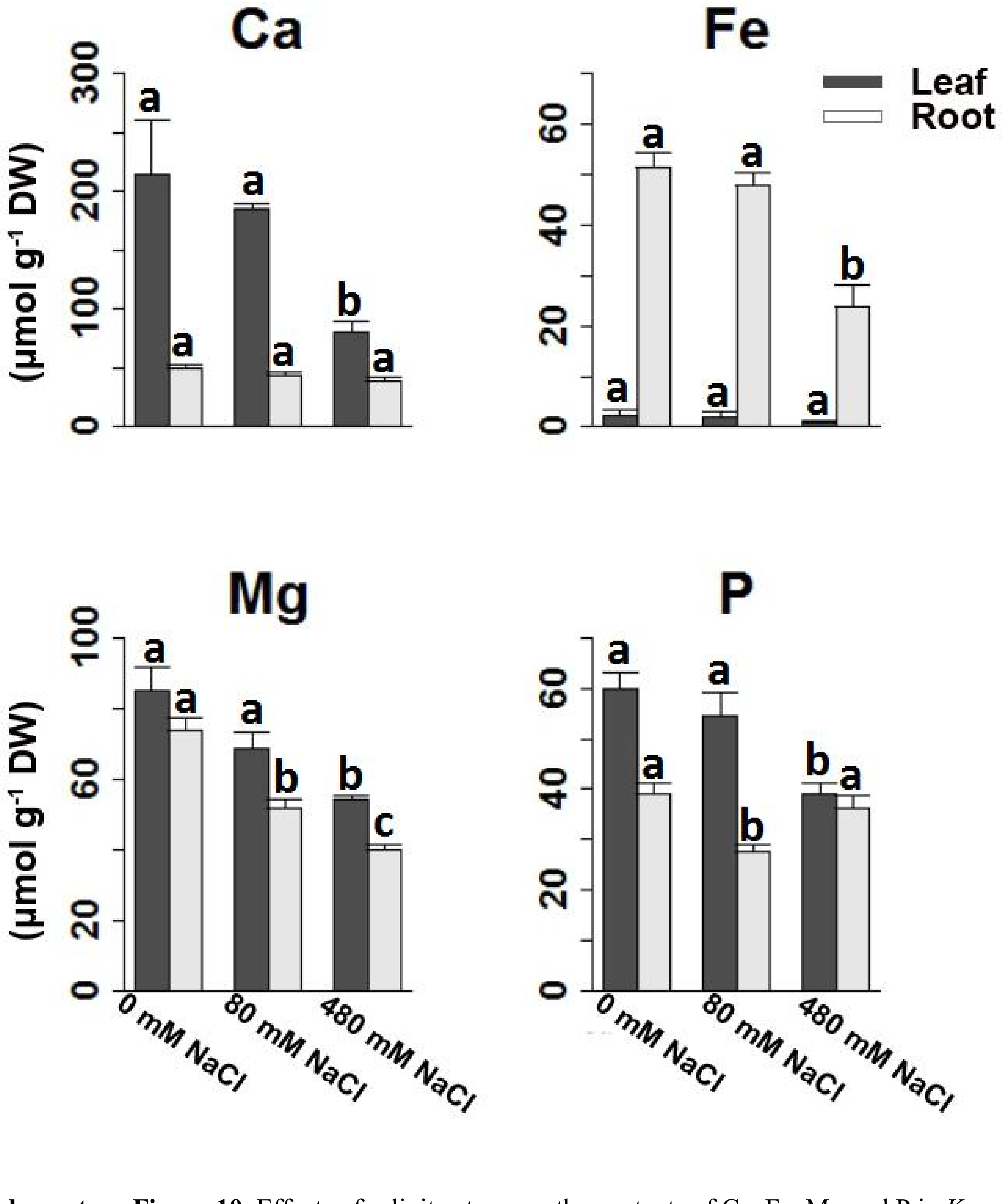

Supplementary Figure 10. Effects of salinity stress on the contents of Ca, Fe, Mg and P in *K. sieversiana*. The 30-d-old seedlings were subjected to 0,80 and 480mM NaCl for 30 d. Different letters above the bars indicate significant differences among treatments according to a least significant difference test (P ≦ 0.05).

Supplementary Table 1. Primer information for the validation of DEGs.

	F	R
UBQ 10	GAAAGCAGTTGGAAGATGGCAGGAC	CGAAGACGCAGAACCAGGTGGAG
TRINITY_DN223354_c1_g1|m.6046	CGGGCATTCTTCAGTGATAGGGG	TCTCGGCTTTATTTCCAGGGGTT
TRINITY_DN224332_c0_g4|m.1276	TTGGAAGGACTTTACTGTCTCACC	CTATTCCGACTTAACCTTGGACG
TRINITY_DN223233_c1_g1|m.16947	TCACCATTACTTATGCCCTCCTT	TGACTACTGCTGGACCGATACTTC
TRINITY_DN225973_c1_g3|m.8014	CGACGGCTGCGTTGACTACTTTG	GCCCGAGCTTCTTCAGGATTACG
TRINITY_DN198904_c0_g1|m.9620	ACCCAAACAAATAATAATGGCTGAA	GAGGAGTCAAATGATGCGAAGTA
TRINITY_DN227624_c1_g4|m.7191	TGGACTTGGGCTTCAGAATGGTA	TAGCGGCTGTATGACTTGTTGTT
TRINITY_DN219148_c0_g1|m.7706	ATGCTTCTCATTAGTCTTATTCGTGC	GACCTTACTACGCCCTCAACTCC
TRINITY_DN224752_c1_g2|m.7735	AAAGGCAATCTGACTGACAATACA	AACACTTATCAACTGGACCTCCC
TRINITY_DN226384_c0_g1|m.2958	AGTTGTGGGACCCGTTGGTTCAT	AAGCCCGGCTTTGCTCGTTTTCT
TRINITY_DN226441_c0_g1|m.777	GATGAAGGGAATAAGTTGTGGCT	AGGATGACCGTCGTATCGTTGTA

Supplementary Table 2. **Overview of unigene annotations.**

	Numbers of unigenes	Percentage of unigenes(%)
**NR**	38,686	82.62
**Swiss-prot**	29,309	62.59
**GO**	3,306	7.06
**KAAS**	6,774	14.47
**COG**	30,464	65.06
**CDD**	14,265	30.47

Supplementary Table 3. KEGG pathway enrichment of *K. sieversiana* unigenes.

	**Pathway ID**	**Pathway**			**DEGs with pathway [illegible]**	**All genes with pathway [illegible]**
**Leaf**	**ko03010**	Genetic Information Processing	Translation	Ribosome	114	240
	**ko00195**	Metabolism	Energy metabolism	Photosynthesis	32	55
	**ko00196**	Metabolism	Energy metabolism	Photosynthesis - antenna proteins	9	15
	**ko00261**	Metabolism	Biosynthesis of other secondary metabolites	Monobactam biosynthesis	6	7
	**ko00860**	Metabolism	Metabolism of cofactors and vitamins	Porphyrin and chlorophyll metabolism	16	41
	**ko00300**	Metabolism	Amino acid metabolism	Lysine biosynthesis	7	11
	**ko00260**	Metabolism	Amino acid metabolism	Glycine, serine and threonine metabolism	20	64
	**ko00710**	Metabolism	Energy metabolism	Carbon fixation in photosynthetic organisms	16	48
	**ko04110**	Cellular Processes	Cell growth and death	Cell cycle	23	85
**Root**	**ko00940**	Metabolism	Biosynthesis of other secondary metabolites	Phenylpropanoid biosynthesis	43	119

**ko00280**	Metabolism	Amino acid metabolism	Valine, leucine and isoleucine degradation	17	51
**ko00073**	Metabolism	Lipid metabolism	Cutin, suberine and wax biosynthesis	10	21
**ko04016**	Environmental Information Processing	Signal transduction	MAPK signaling pathway - plant	25	105
**ko00250**	Metabolism	Amino acid metabolism	Alanine, aspartate and glutamate metabolism	12	37
**ko00052**	Metabolism	Carbohydrate metabolism	Galactose metabolism	12	38
**ko00196**	Metabolism	Energy metabolism	Photosynthesis - antenna proteins	7	15
**ko00270**	Metabolism	Amino acid metabolism	Cysteine and methionine metabolism	18	74
**ko00260**	Metabolism	Amino acid metabolism	Glycine, serine and threonine metabolism	16	64
**ko00710**	Metabolism	Energy metabolism	Carbon fixation in photosynthetic organisms	13	48
**ko00071**	Metabolism	Lipid metabolism	Fatty acid degradation	11	40
**ko00910**	Metabolism	Energy metabolism	Nitrogen metabolism	8	25
**ko00195**	Metabolism	Energy metabolism	Photosynthesis	13	55
**ko00430**	Metabolism	Metabolism of other amino acids	Taurine and hypotaurine metabolism	4	7

**ko04075**	Environmental Information Processing	Signal transduction	Plant hormone signal transduction	27	159
**ko03320**	Organismal Systems	Endocrine system	PPAR signaling pathway	8	28
**ko00500**	Metabolism	Carbohydrate metabolism	Starch and sucrose metabolism	18	94

Supplementary Table 4. Validation of DEGs by real-time quantitative PCR.

ID	R^2	P
TRINITY_DN223354_c1_g1|m.6046	0.89	0.017
TRINITY_DN224332_c0_g4|m.1276	0.7	0.118
TRINITY_DN223233_c1_g1|m.16947	0.87	0.024
TRINITY_DN225973_c1_g3|m.8014	0.84	0.036
TRINITY_DN198904_c0_g1|m.9620	0.96	0.002
TRINITY_DN227624_c1_g4|m.7191	0.79	0.062
TRINITY_DN219148_c0_g1|m.7706	0.92	0.008
TRINITY_DN224752_c1_g2|m.7735	0.97	0.002
TRINITY_DN226384_c0_g1|m.2958	0.98	0.001
TRINITY_DN226441_c0_g1|m.777	0.68	0.138

Supplementary Table 5. Effects of salinity stress on expression of ion compartmentation genes. The 30-d-old seedlings were subjected to 0, 80 and 480mM NaCl for 2 d.

Genes ID	Leaf (FPKM)			Root (FPKM)			Genes family
	0 mM NaCl	80 mM NaCl	480 mM NaCl	0 mM NaCl	80 mM NaCl	480 mM NaCl	
**TRINITY_DN216785_c2_g2|m.13772**	1.75 ± 0.06[a]	1.60 ± 0.05[a]	5.52 ± 0.78[a]	30.08 ± 1.39[a]	34.92 ± 1.57[a]	40.84 ± 0.41[a]	**NHX**
**TRINITY_DN218503_c0_g2|m.12824**	1.50 ± 0.08[a]	1.99 ± 0.22[a]	4.08 ± 0.33[a]	13.48 ± 0.43[a]	15.58 ± 0.84[a]	15.77 ± 0.37[a]	**NHX**
**TRINITY_DN226373_c0_g2|m.5681**	67.92 ± 6.20[a]	83.86 ± 15.37[a]	102.56 ± 7.12[a]	21.56 ± 1.05[a]	35.18 ± 3.05[a]	39.12 ± 1.06[a]	**NHX**
**TRINITY_DN218402_c0_g2|m.2511**	122.03 ± 12.64[a]	143.31 ± 19.37[a]	81.01 ± 7.17[a]	15.44 ± 0.40[c]	21.11 ± 1.16[b]	33.30 ± 1.55[a]	**HA**
**TRINITY_DN228495_c2_g2|m.162**	171.02 ± 9.57[a]	220.40 ± 23.87[a]	146.49 ± 10.62[a]	533.17 ± 64.77[a]	432.29 ± 27.62[a]	434.73 ± 26.10[a]	**AVP1**
**TRINITY_DN220826_c3_g1|m.7289**	1.92 ± 0.40[a]	2.11 ± 0.41[a]	3.82 ± 1.35[a]	107.98 ± 16.78[a]	98.05 ± 8.77[a]	118.90 ± 18.16[a]	**AVP1**

frontiers
